# Grief, bereavement and prolonged grief disorder: scoping and mapping the evidence

**DOI:** 10.1192/bjo.2025.10050

**Published:** 2025-07-11

**Authors:** Gary Raine, Claire Khouja, Meena Khatwa, Helen Fulbright, Katy Sutcliffe, Amanda J. Sowden

**Affiliations:** Centre for Reviews and Dissemination, University of York, UK; EPPI-Centre, Social Science Research Unit, UCL Institute of Education, University College London, UK

**Keywords:** Grief, bereavement, prolonged grief disorder, scoping review, evidence map

## Abstract

**Background:**

Some individuals experience abnormally persistent and intense symptoms of grief that significantly interfere with daily functioning. This condition has been described using terms such as complicated or prolonged grief and prolonged grief disorder (PGD).

**Aims:**

To identify the availability of evidence addressing a range of policy relevant issues related to grief, bereavement and PGD. In this paper we focus on the availability of evidence from systematic reviews.

**Method:**

We searched 12 databases and the websites of 18 grief- or bereavement-related organisations. Using key characteristics extracted from included reviews, we produced a high-level overview of the available evidence that enabled potential research gaps to be identified.

**Results:**

We identified 212 reviews – 103 focused on people’s experiences of grief/bereavement including service use; 22 reported on PGD prevalence, 42 on PGD risk factors, 37 on factors that influence grief more broadly and 80 on the effectiveness of grief-related interventions. Fifty-five reviews focused on multiple issues of interest. Half of reviews focused on a specific cause/type of death (*n* = 108). Of these reviews, most focused on three main causes/types of death: a specific health condition or terminal illness (*n* = 36), perinatal loss (*n* = 34) and suicide (*n* = 20).

**Conclusions:**

We identified a large number of reviews, but key evidence gaps exist, particularly in relation to intervention cost-effectiveness and social, organisational or structural-level interventions that are needed for addressing inequities and other modifiable factors that can impair grieving and potentially increase the risk of PGD.

Grief is a natural reaction to bereavement, and most people adapt successfully to their loss over time without the need for formal support or treatment.^1,2^ Some individuals experience abnormally persistent and intense symptoms of grief that significantly interfere with daily functioning and increase the risk of adverse physical and mental health outcomes.^2–4^

This condition has been described using various terms including chronic, complicated, disordered, pathological, prolonged, morbid and traumatic grief; persistent complex bereavement disorder; and, most recently, prolonged grief disorder (PGD).

PGD has recently been recognised as a distinct mental health disorder in both ICD-11 and DSM-5-TR, published by the World Health Organization^5^ and American Psychiatric Association, respectively.^6^ A key diagnostic criterion of PGD in both classification systems is that the duration of an individual’s grief exceeds expected social, cultural or religious norms.^7,8^ However, the criteria for PGD in ICD-11 and DSM-5-TR are not identical. For example, DSM-5-TR requires the death of an adult to have occurred at least 12 months prior to diagnosis compared with at least 6 months in ICD-11.^9^ The classification of PGD as a mental health disorder has not received universal approval. Various concerns have been raised around issues such as pathologising and medicalising grief, stigmatisation of the bereaved and increased use/misuse of medication.^10–12^

The prevalence of PGD among bereaved adults as a result of non-violent loss is estimated to be approximately 10%.^13^ However, prevalence varies and PGD is thought to be more common in certain circumstances – for example, following the death of a partner or child, and when individuals are bereaved as a result of a sudden, unexpected or violent death, including suicide.^2,14–16^

Social and structural inequities make individuals more susceptible to poorer health outcomes by constraining agency and health-related behaviour, as well as by creating barriers to care.^17–21^ Consequently, death, loss and bereavement are disproportionally experienced by structurally vulnerable groups.^20,22^ The combined life circumstances of these individuals may result in greater vulnerability to PGD. Notably, social and structural factors, including lower socioeconomic status and low income, have been linked to a higher risk of developing the condition.^9,23^ In addition, research from the UK has found that individuals on a low income face greater difficulties accessing services following bereavement and are also more reluctant to seek support.^24,25^

In 2022, the UK Commission on Bereavement published a review of bereavement support in England and other countries of the UK.^26^ The commission gathered evidence from different stakeholder groups to improve understanding of the challenges faced by bereaved individuals and identify barriers to accessing support. Inequality in access to appropriate formal support was highlighted as a key issue, and certain groups were reported to be particularly poorly served. The review also detailed a range of social, organisational and structural factors that impact negatively on people’s experience of bereavement and act as barriers to support. These included stigma associated with certain types of deaths and with seeking professional support; inadequate support in the workplace; negative interactions with organisations with which individuals come into contact following a bereavement; and inadequate funding and coordination of services. Some respondents raised the issue of prolonged grief and gave examples of factors thought to increase the risk of it developing. The commission produced eight key principles and related recommendations for change, which included having an increased focus on tackling inequality in the provision of bereavement services. It also called for more research, particularly in relation to better understanding the needs of the bereaved, improving services for underserved groups and evaluating bereavement support.^26^

This paper reports on a systematic scope of the evidence on grief, bereavement and PGD commissioned by the Department of Health and Social Care (DHSC) in England. Scoping reviews are widely used for summarising and mapping the extent of evidence on a particular topic and identifying potential research gaps.^27^ We aimed to identify the availability of evidence from systematic reviews on the following issues:

- the extent and nature of PGD;
- risk factors for PGD;
- grief and bereavement experiences including the needs of the bereaved; use of services and equity in relation to access;
- the effectiveness of interventions for preventing or treating PGD.

We also sought to identify protocols for ongoing reviews, as well as primary studies, that reported on the types of bereavement support services that are available in the UK. Our goal was to produce a high-level overview (‘map’) of the available evidence to enable identification of potential research gaps. In this paper, we focus on the availability of evidence from published systematic reviews and ‘reviews of reviews’ only. We did not seek to extract, evaluate and synthesise findings from included reviews, protocols or primary studies. Please see our full project report^28^ for an overview of review protocols and findings on the availability of evidence from primary studies, which can be accessed from https://eppi.ioe.ac.uk/cms/Default.aspx?tabid=3929.

## Method

A protocol for our scope of the literature was approved by the DHSC but was not registered on the International Prospective Register of Systematic Reviews (PROSPERO), because scoping and mapping reviews are not eligible for inclusion in the registry. We searched the following 12 academic databases on 28 October 2022: MEDLINE (OVID); Embase (OVID); PsycINFO (OVID); CINAHL (EBSCO); Cochrane Database of Systematic Reviews (CDSR) (Wiley); Epistemonikos (https://www.epistemonikos.org/en); Health Management Information Consortium (HMIC) (OVID); Social Policy & Practice (OVID); Social Care Online (https://www.scie.org.uk/social-care-online/); Social Services Abstracts (ProQuest); EThOS (British Library); and PROSPERO (CRD). Searches were date limited from 2015 onwards to maximise the relevance of the evidence identified. No language restrictions were applied. The search strategy was developed in Ovid MEDLINE by an information specialist and consisted of broad terms for grief and bereavement. The MEDLINE search strategy was subsequently adapted for use with the other academic databases. We also searched the websites of 18 key organisations related to grief and bereavement, which included the Center for Complicated Grief (Columbia University), National Bereavement Alliance, Sue Ryder, Marie Curie Palliative Care Research Centre and the Caresearch Project: Palliative Care Knowledge Network. The MEDLINE search strategy and a complete list of organisation websites that we searched are available in Supplementary file 1.

We uploaded records identified from database searches into EPPI-Reviewer software.^29^ A sample of 40 records was pilot screened by three reviewers to ensure consistency in screening decisions. The remaining records were screened by one reviewer only. The full texts of potentially relevant reviews were screened independently by two reviewers.

Records were selected for inclusion in the evidence map based on clearly defined inclusion criteria, which are provided in full in Supplementary file 1. In summary, we included systematic reviews focusing on any bereaved population that reported on one or more of the following: the frequency or risk of PGD; the relationship between grief-related outcomes and potential risk factors for PGD; experiences of grief/bereavement, including use of bereavement services and the needs of the bereaved; and intervention effectiveness/impact, cost-effectiveness and/or implementation. For each included publication, key characteristics were extracted by one reviewer and checked by a second. This information was used to produce a high-level descriptive summary that detailed the extent and nature of the current evidence base.

## Results

**Fig. 1:**
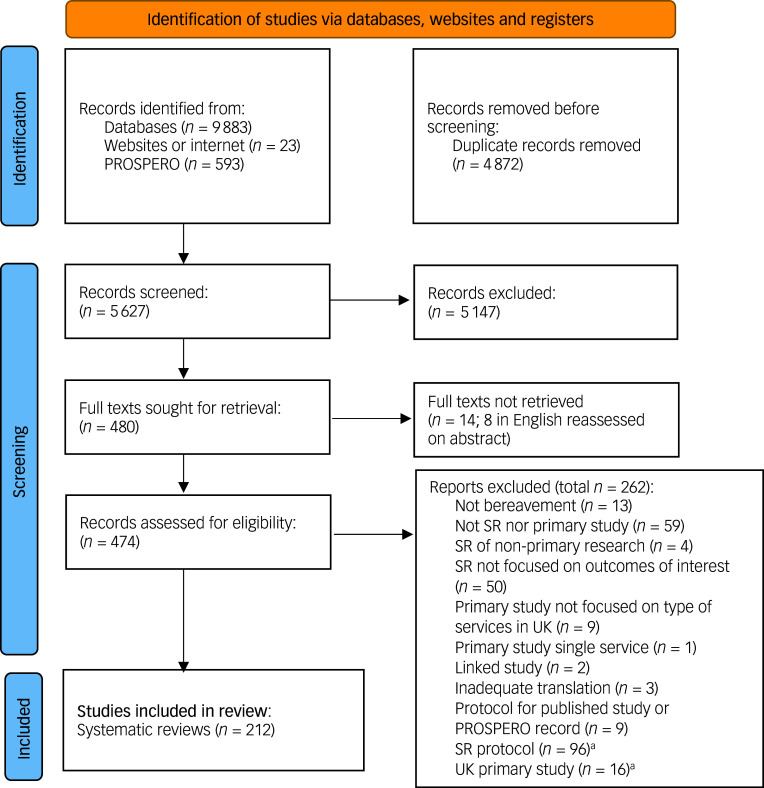
Flow of studies through the review. SR, systematic review; PROSPERO, International Prospective Register of Systematic Reviews. a. SR protocols and UK primary studies were included in the published report and interactive map, but not in this paper.

We screened 5627 records and included 212 systematic reviews. The flow of studies through the review is shown in Fig. 1. An interactive map of all included publications (i.e. including the protocols and primary studies not reported on in this paper) is available from https://eppi.ioe.ac.uk/eppi-vis/login/open?webdbid=471.

In the following sections, we present an overview of the key characteristics of included reviews. Full details about included reviews are provided in Supplementary file 2. Please see the full project report for a more detailed description of the evidence for each of the main issues of interest.

### Overview of included reviews (2015–2022)

#### Year of publication

Sixty-one per cent of reviews (*n* = 129) were published between 2020 and 2023, and over three-quarters were published from 2018 (78%, *n* = 166).

#### Topic focus of the reviews

To assess the extent of evidence available on the key issues of interest, we coded reviews according to the broad topic on which they focused. The largest group of reviews explored individuals’ grief and bereavement experiences (*n* = 103).^30–132^ This group encompassed reviews that addressed one or more of the following issues: the use of formal or informal sources of support, barriers to accessing support, equity related to grief/bereavement and the use of services, coping with grief, post-bereavement needs, and views about the factors that facilitate or impair the grieving process. One of the 103 reviews was a review of reviews.^94^ The second largest group of reviews focused on the effectiveness of grief-related interventions (*n* = 80).^33,58,67,73–75,82,88,89,94,96,118,133–200^ This included three reviews of reviews.^94,137,190^ Forty-two reviews addressed risk and protective factors for prolonged grief, correlates of PGD or factors moderating its prevalence.^13,32,38,71,116,118,130,168,172,175,178,187,191,201–229^ We also identified 37 reviews reporting on what we called ‘general grief reactions’. These reviews examined the relationship between specific factors and outcomes such as the severity or intensity of grief and/or individual adjustment after bereavement, rather than focusing on prolonged grief specifically.^31,33,38,42,50,55,63,67,69,71,72,76,90,99,115,116,124,130,187,191,202–204,209,212,213,215,220,221,224,226,230–235^ We identified 22 reviews focused on the extent and nature (prevalence) of prolonged grief among various groups of bereaved people.^13,71,76,116,175,178,203,205,208,212,216–218,220,222,228,229,236–240^ Fifty-five out of the 212 reviews focused on two or more issues of interest. Table 1 provides a summary of all reviews by focus and key characteristics.

**Table 1:** Number of reviews by focus and key characteristics

Characteristics	Extent of PGD	Risk factors for PGD	General grief	Experiences^a^	Intervention effectiveness^b^	All reviews
Relationship						
Specific	7	14	22	74	42	123
Any family/friends	4	13	7	11	9	31
Other non-specific	11	15	8	18	29	58
All non-specific	15	28	15	29	38	89
Personal characteristics						
Adults^c^	13	21	12	15	24	61
Children^c^	1	3	6	7	7	16
Female		1		5	4	9
Male			1	4		4
LGBT+				2		2
Disability	1	1		1	1	2
Minority ethnic			1	3		3
Refugees/migrants	2	1	1	1		3
Substance misuse	1	1			1	1
Structural vulnerability				1		1
Country						
UK, USA and other high-income	2	3	4	14	7	20
LMIC				2	1	2
Low-income and sub-Saharan Africa		1		3	1	3
HMIC				1		1
China, Hong Kong or Taiwan	2	1	1	1		3
Asia-Pacific				1		1
Cause/type of death						
Specific health issue/terminal illness	6	9	6	14	15^d^	36
Perinatal		2	2	23	13	34
Suicide		1	3	13	8	20
Violent/unnatural	2	4	1	2	2	7
Assisted suicide		2	1	3		3
Sudden/unexpected				1	1	2
Non-violent	2	1				2
Drug related				2		2
COVID-19					1	1
Mass events – illness or terrorism, etc.					1	1
No cause of death: specific setting^e^						
Hospital settings		2		8	6	15
Long-term care			1	1		1
No specific cause of death or setting	12	21	23	36	33	88
**Totals**	**22**	**42**	**37**	**103**	**80**	**212**

PGD, prolonged grief disorder; LGBT+, lesbian, gay, bisexual and transgender; LMIC, low- and middle-income countries; HMIC, high- and middle-income countries. Some reviews reported findings on multiple issues, and some included both primary studies and reviews.

a. Includes one review of reviews.

b Includes three reviews of reviews.

c Based on inclusion criteria. Other reviews may also have reported only findings from studies of adults owing to a lack of eligible child-focused research.

d One review focused on deaths due to medical illness and unforeseen circumstances but was included only in the specific health condition category.

e Excludes reviews focused on both a specific cause and setting.

### Characteristics of the bereaved (*n* = 212)

#### Relationship to the deceased

A majority of reviews (*n* = 123) focused on bereaved individuals with a specific relationship to the deceased (carer, parent, spouse/partner, sibling, son/daughter, grandparent, health or care professional or work colleague). The remaining 89 reviews either had a generic focus on ‘family’ or ‘family and friends’ (*n* = 31) or focused on bereaved individuals with no specified relationship to the deceased (*n* = 58) (Table 1).

Table 2 shows the specific relationship of bereaved individuals to the deceased. Over half of the 123 reviews included a focus on bereaved parents (*n* = 64). Forty-six focused on parents, mothers or fathers only, 15 focused on a defined group of parents with other family members, and 3 focused on both parents and health professionals. Fifty-nine reviews focused on a range of other relationships: informal/family carers (*n* = 18), child (parent loss) or sibling (sibling loss) (*n* = 13), individuals with a professional relationship to the deceased or relatives (*n* = 11), the spouse or partner of the deceased (*n* = 10), children and their surviving parent/carer (*n* = 3), work colleagues (*n* = 1) and daughters following the death of their mother (*n* = 1). The remaining two reviews focused on a combination of individuals: siblings, extended family and members of the community (*n* = 1) and co-workers, family members and close friends (*n* = 1).

**Table 2:** Specific relationship to the deceased

Relationship to the deceased	Extent of PGD	PGD Risk factors	General grief	Experiences	Intervention effectiveness	All reviews
Parents only	2	3	5	21	10	34
Mothers		1		4	4	8
Fathers			1	4		4
Parents with other family				11	9	15
Parents and professionals				3		3
Informal carers	3	7	4	6	8	18
Child/sibling	1	2	5	5	5	13^a^
Professionals			2	11		11
Spouse/partner	1		4	6	3	10
Child and surviving parent/carer		1	1	1	1	3
Co-worker/colleague				1		1
Daughters (death of mother)				1		1
Siblings, extended family or community					1	1
Co-workers, family members or close friends					1	1
**Totals**	**7**	**14**	**22**	**74**	**42**	**123**

PGD, prolonged grief disorder.

a. Three of the 16 reviews that included only children/young people did not focus on individuals with a specific relationship to the deceased.

Numbers in the five topic focus columns do not add up to the ‘All reviews’ total because some reviews reported on multiple topics.

#### Relationship to the deceased and review focus

Most reviews addressing PGD prevalence (15/22) and PGD risk factors (28/42) had a generic focus on ‘family’ or ‘family and friends’, or focused on bereaved individuals with no specified relationship to the deceased. In contrast, a majority of reviews reporting grief/bereavement experiences (74/103), intervention effectiveness (42/80) and general grief reactions (22/37) focused on individuals with a specific relationship to the deceased. Of the 116 reviews examining experiences and/or intervention effectiveness that focused on individuals with a specific relationship to the deceased, the majority (40/74 reviews of experiences and 23/42 intervention reviews) focused on parents (including mothers or fathers) or a defined group of parents with other family members. A further three reviews of experiences focused on parents and health professionals. Informal carers were the focus of half of the 14 reviews exploring PGD risk factors among individuals with a specific relationship to the deceased (*n* = 7). The largest number of reviews on PGD prevalence among individuals with a specific relationship to the deceased also focused on informal carers (3/7). Full details of the relationship to the deceased by review focus are provided in Table 2.

#### Reviews focused on bereaved individuals with other specific characteristics

Ninety-four reviews focused on bereaved individuals with other specific characteristics. Of these, 61 restricted inclusion to bereaved adults and 16 had a primary focus on bereaved children and young people. Nine reviews focused solely on females and four had a focus on males only. Twelve reviews focused on marginalised or minority populations. Three of these 12 reviews focused on minority ethnic groups; 3 on refugees, migrants or asylum seekers; 2 on lesbian, gay, bisexual and transgender (LGBT+) individuals; and 2 on individuals with an intellectual disability. Another review comprised studies of individuals who misused drugs and one focused on those positioned as structurally vulnerable in bereavement. Eight reviews focused on individuals with multiple specific characteristics, such as adults from a minority ethnic background or adult refugees.

### Reviews with geographical restrictions

Thirty of the 212 reviews applied geographical restrictions. Two-thirds of these reviews focused on studies conducted in the UK, USA or other high-income countries (*n* = 20). The remaining 10 focused on low-income countries including in sub-Saharan Africa (*n* = 3), China, Hong Kong or Taiwan (*n* = 3), low- or middle-income countries (*n* = 2), high- or middle- income countries (*n* = 1) and the Asia-Pacific region (*n* = 1).

### Reviews focused on a specific cause/type of death

**Fig. 2:**
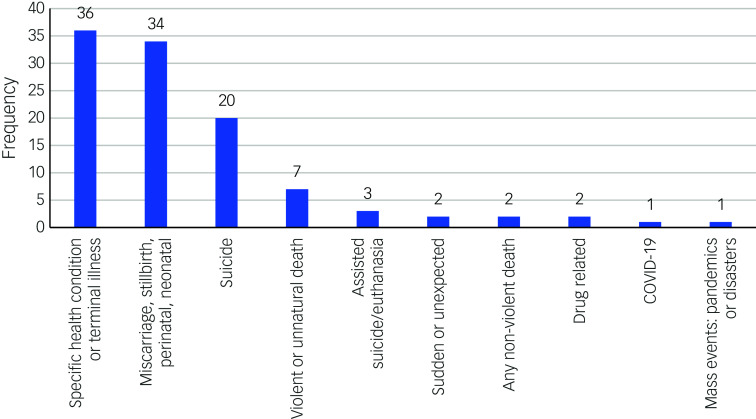
Reviews focused on a specific cause/type of death.

Approximately half of included reviews (108/212) focused on a specific cause or type of death. Figure 2 shows the cause or type of death reported in reviews.

Of the 108 reviews, 90 focused on 3 main causes or types of death: a specific health condition or terminal illness (e.g. cancer or dementia), excluding COVID-19 deaths (*n* = 36), perinatal loss (miscarriage through to neonatal death, including stillbirth [*n* = 34]), or suicide (*n* = 20). The other 18 reviews examined violent or unnatural deaths (*n* = 7), assisted suicide or euthanasia (*n* = 3), sudden and unexpected deaths (*n* = 2), non-violent deaths (*n* = 2), drug-related deaths (*n* = 2), COVID-19-related deaths (*n* = 1) and mass events such as pandemics, natural and human-made disasters, or terrorism (*n* = 1).

Sixteen reviews did not examine a specific cause or type of death but were focused on a specific setting: intensive/critical care (*n* = 9), acute care settings (*n* = 3), in-hospital settings (*n* = 2), emergency department (*n* = 1) or long-term care (*n* = 1). Eighty-eight reviews did not focus on a specific cause or type of death or deaths in a specific setting. A breakdown of cause/type or setting of death by review focus is provided in Table 1. An additional breakdown of cause/type or setting of death by relationship to the deceased is provided in Supplementary file 3.

### Reviews on intervention effectiveness

Over half of reviews (43/80) that reported on intervention effectiveness had a focus on any type of bereavement support, most commonly for specific population groups and/or following specific causes/types of death: for example, any form of intervention for bereaved children or parents or interventions for individuals bereaved by suicide. This included reviews focused on any bereavement support or services in medical or palliative care settings (*n* = 6), as well as those delivered by healthcare professionals (*n* = 3).

Thirty-seven reviews had a focus on specific types of intervention. This included multiple reviews that examined the effectiveness of psychological-based interventions (*n* = 9), psychosocial interventions (*n* = 5), internet-based interventions (*n* = 4), peer support (*n* = 3) and bereavement support (individual/group support), or specifically, structured/formal bereavement support including suicide postvention services (*n* = 3). Three reviews focused on multiple intervention types, two of which involved psychological interventions delivered online, and one on online peer support. One other review focused on psychosocial or psychotherapeutic interventions. A range of other types of support were the primary focus of 11 reviews: brief contact interventions, community-based support, system-level approaches, physical activity-based interventions, interventions based on the Dual Process Model of Coping, those that incorporate rituals and ritualised acts, use of intensive care unit diaries by bereaved relatives, parental contact with their baby following stillbirth, bereavement groups; bereavement follow-up and memory making in end-of-life care. Four reviews also evaluated specific types of therapy: dignity therapy, visual art therapy, acceptance and commitment therapy, cognitive behavioural therapy and mindfulness therapy.

### Reviews on equity issues in relation to bereavement and service access and use

A secondary objective of our scoping review was to identify evidence on equity issues in relation to bereavement and access to support services. We examined in greater depth five reviews that focused on the experiences of marginalised or minority populations, and report findings related to access to, or use of, support services.^37,41,64,69,93^ Three of the reviews had a key focus on equity issues.^37,41,93^

The review by Mayland et al examined bereavement care in the UK for ethnic minority communities. It identified barriers and facilitators to accessing care, explored satisfaction with service provision and investigated whether any models of care provision exist for addressing the bereavement-specific needs of ethnic minority communities.^93^ Bindley et al examined the existing literature on bereavement and structural vulnerability. They reported evidence on the way in which unequal social status, related to gender, class, sexuality, ethnicity and age, influences access to, and use of, bereavement support and interactions with institutions.^37^ The review by Bristowe et al reported evidence on the barriers and stressors experienced by LGBT+ individuals in accessing support following the loss of a partner.^41^

The other two reviews also reported some relevant findings related to service access or use, both of which were focused on members of the Latino community predominately in the USA.^64,69^ These reviews examined factors, such as cultural values, that are potentially important in terms of relationships with health care providers, service provision and help-seeking from formal services. One of the reviews also reported findings on individuals’ perceptions and satisfaction with services.^64^

We also identified nine reviews that explored gendered experiences of grief and bereavement.^48,57,75,80,85,95,99,125,128^ Seven of these reviews explored experiences following perinatal loss, including miscarriage and stillbirth, of which four focused on mothers only^48,75,85,128^ and three on fathers only.^80,99,125^ Another review explored the experiences of fathers following the death of a child under 21 years old, but excluded studies of miscarriage and stillbirth.^95^ The remaining review focused on daughters’ experiences of maternal bereavement.^57^ Six of the nine reviews reported findings on individuals’ experiences of using health services.^48,75,80,85,99,125^

## Discussion

This paper provides a high-level overview of the available evidence related to grief, bereavement and PGD identified from 212 systematic reviews published up to October 2022. Our work reveals that issues related to grief and bereavement, including PGD, have been extensively researched, but key evidence gaps were identified and these are discussed below. In November 2024, we conducted a supplementary search of six databases from October 2022 onwards to check whether the gaps in evidence remained. The search retrieved 542 records, which we screened purposively for any reviews that addressed the identified evidence gaps only. Findings from this supplementary search were incorporated into the Discussion. Further details about this search and the strategy used are detailed in Supplementary file 1.

When categorising review focus for our map, consideration was given to the context and aim of each review, as well as to the findings reported. There exists a degree of overlap between categories in terms of the nature of reported findings. For example, the categories of grief/bereavement experiences and intervention effectiveness both included some reviews that reported qualitative findings on people’s perceptions of services and the benefits/impacts of specific interventions.

Reviews reported PGD prevalence estimates for a broad range of bereaved population groups and causes of death. These often focused on individuals who are potentially more vulnerable to developing grief-related problems, such as bereaved parents and following various types of death including violent deaths, suicides and deaths from cancer. Most reviews reporting PGD prevalence also explored factors that potentially influence the risk of developing the disorder.

Reviews have investigated the relationship between prolonged grief and a broad range of factors. However, without a more in-depth analysis of the specific factors assessed in existing research, it remains unclear to what extent reviews have gone beyond a focus on individual- and interpersonal-level factors to consider broader social, organisational, cultural and structural factors that potentially influence the risk of developing PGD.

There is a large body of literature on individuals’ experiences of grief and bereavement. Included reviews reported a broad range of experiences, especially in terms of population groups and types of death. However, across reviews of experience there was a predominant focus on bereaved parents and other family members following the loss of a child (*n* = 43), particularly in the perinatal period (*n* = 23). We also identified a sizeable number of reviews focused on experiences following common causes of bereavement including suicide (*n* = 13), and health conditions such as dementia, cancer and other terminal illnesses (*n* = 14).

Reviews of individuals’ grief and bereavement experiences are potentially valuable for informing policy and practice within the context of minimising PGD. These may facilitate a better understanding of the grieving process and what helps people cope effectively with grief, as well as informing the provision of services and the development of interventions. Individual focused interventions will not be sufficient on their own to reduce the prevalence of prolonged grief on a population level. Evidence on individuals’ lived experience of grief and bereavement could be useful for informing organisational change and other preventative efforts intended to address broader social and structural factors that disrupt the process of grieving.

It is important to consider issues of equity in relation to bereavement, particularly within the context of reducing health disparities. We identified three published reviews with a key focus on equity issues relating to bereavement and service provision. These reviews explored the experiences of ethnic minority communities in the UK,^93^ LGBT+ individuals^41^ and those considered structurally vulnerable in bereavement.^37^ Notably, Mayland et al reported an overall lack of research on bereavement services in the UK for individuals from ethnic minority communities, and highlighted a need to strengthen the evidence base.^93^ Similarly, the review by Bristowe et al on the bereavement experiences of LGBT+ communities was limited by a scarcity of research conducted with bereaved bisexual and trans people. They cautioned that the use of the term LGBT to describe research can be misleading when some groups are significantly under-represented in the sample.^41^

The review by Bindley et al, published in 2019 on social and structural inequity in bereavement, identified only four studies that were focused on issues related to income, employment and financial circumstances, and three of these were conducted prior to 2012.^37^ This represents an important gap in the literature, especially considering the difficulties in accessing services following bereavement reported by individuals on a low income and their greater reluctance to seek support.^24,25^ We are aware of one qualitative study published in 2022 which found that housing insecurity can negatively impact bereavement and the process of grieving among low-income communities in the UK.^241^ There may be other recently published studies examining the influence of broader socioeconomic determinants and inequities on post-bereavement needs, experience and service use that have not yet been incorporated into evidence reviews. Additional work to identify and synthesise the findings from such studies could be beneficial for informing policy and practice.

From our supplementary search conducted in November 2024, we identified one recently published review reporting the bereavement experiences of individuals who were homeless.^242^ Nonetheless, the literature would benefit from a greater focus on the bereavement needs and experiences of the most marginalised and disadvantaged communities as these individuals, in particular, may have difficulty accessing support and be at higher risk of PGD.^9,19,20,23–25^ This includes people with a range of disabilities, gypsy, Roma and traveller communities, refugees and asylum seekers, and trans individuals.

In terms of issues related to gender and equity, we identified five published reviews exploring the bereavement experiences of women only and four focused solely on men. All but one of these reviews focused on women as mothers or men as fathers following the death of a child or a miscarriage. Even the one study not focused on bereaved parents explored the experiences of daughters following the death of their mother. The lack of reviews of gendered research that go beyond a focus on parental experiences, or a limited type of child–parent relationship, represents another notable evidence gap. Applying a broad gendered lens to the examination of bereavement experiences including service use is important given that some studies have identified a relationship between being a woman and experiencing PGD.^9,210^ There is also evidence that men are less likely to seek help and use services when experiencing mental health difficulties,^243^ which could also include grief-related problems. Gender socialisation is recognised to play a key role in the responses of men and women to bereavement.^244,245^ We identified one additional review of eight studies from our supplementary search examining men’s bereavement experiences, which did include some studies of grief following deaths in non-parental relationships: for example, experiences following the loss of a spouse or military colleague.^246^ Notably, the review authors highlighted limitations with their search strategy, and they only searched a small number of databases; consequently, it is unlikely that the review included all relevant studies.

When considering issues related to bereavement and equity in the provision of support, it would be particularly beneficial for research to adopt an intersectional focus in order to better understand how social characteristics such as gender, ethnicity and disability interact with wider social and structural inequities to influence individuals’ bereavement experiences, access to support and use of services.^247^

Reviews have evaluated a wide range of different grief-related interventions, but there is a paucity of evidence concerning social, organisational or structural-level interventions. One review did explore system-level responses to bereavement support following major disaster events, but all included studies were focused on the individual level.^157^ Our supplementary search identified a recently published review that explored best practices for supporting mental health clinicians in the military following the death of a patient by suicide.^248^ It reported a range of recommendations for postvention identified from both empirical and non-empirical literature, which included actions on an organisational level. By specifically targeting inequities and other modifiable factors that increase the risk of impaired grieving and PGD before individuals develop problematic symptoms, social/organisational/structural-level interventions potentially have an important role in terms of prevention. We did not identify any reviews focused on the cost-effectiveness of interventions from either our original or supplementary search.

### Strengths and limitations

Our scoping of the evidence was conducted using systematic methods that included comprehensive searching, clearly defined inclusion criteria and systematic coding of key characteristics. We captured a range of key information about each included review, including details on its focus, the participants in included studies and, where applicable, the nature of the intervention being evaluated. Our evidence map is limited by the quality of reporting on included studies by review authors. For example, some key details about included studies may have been omitted or reported inaccurately by review authors.

We identified multiple reviews with a similar topic focus and study aim, particularly in relation to bereavement resulting from perinatal death, terminal/chronic illness or suicide. Consequently, there could exist considerable overlap in the primary studies included across these reviews. When the same study or studies are included in multiple reviews, it can offer reassurance that individual reviews were conducted in a consistent manner and that their results reflect the existing literature. However, study overlap may result in an overestimation of the size and strength of the evidence base.^249,250^

Our main findings are based on reviews published up to October 2022 only; relevant reviews published after this time will not be in our map. However, this is not a major limitation because we did not seek to synthesise findings in order to identify key themes or draw conclusions about intervention effectiveness.^251^ A particular strength of our work is that it illuminates those topics within the field of grief and bereavement that have already been the focus of multiple reviews, and it identifies key gaps in knowledge in other areas, which is potentially valuable for informing decisions about the need for new systematic reviews. By highlighting those topics that have already been extensively reviewed, this work can help prevent research waste resulting from the unnecessary duplication of reviews. This is an issue that has been identified as a significant problem in the field of evidence synthesis.^252^

Our supplementary search indicated that the key evidence gaps identified from our October 2022 search remain. This was unsurprising because our examination of protocols, detailed in the full project report,^28^ indicated that reviews ongoing during the course of our work largely focused on populations/relationships, causes of death, settings or intervention types very similar to existing published reviews.

This work facilitates the identification of evidence gaps across a broad research field; researchers seeking to conduct reviews in this area can begin by exploring evidence gaps and conducting searches to identify whether there have been any newly published reviews that address topics of potential interest, rather than needing to focus on the evidence base in its entirety. Our online interactive map will be of benefit to practitioners, researchers and policymakers because it enables users to quickly locate existing evidence and offers access to additional information about each included review.

To conclude, our goal was to map the available evidence on grief, bereavement and PGD, and to identify gaps in the literature. We found a large number of reviews addressing the extent and nature of PGD; risk factors for the disorder; grief and bereavement experiences; and/or the effectiveness of interventions for preventing or treating PGD. However, the current evidence base is limited by key gaps in the literature, particularly in relation to the cost-effectiveness of interventions, and interventions for addressing social, organisational or structural-level factors that can impair the grieving process and potentially increase the risk of PGD.

## Supporting information

Raine et al. supplementary material 1 Raine et al. supplementary material

Raine et al. supplementary material 2 Raine et al. supplementary material

Raine et al. supplementary material 3 Raine et al. supplementary material

## Data Availability

Data availability is not applicable to this article because no new data were created or analysed in this study.

## Supplementary material

The supplementary material can be found online at https://doi.org/10.1192/bjo.2025.10050
