# Supplementary material for: Grief, bereavement and prolonged grief disorder: scoping and mapping the evidence
Source: BJPsych Open. 2025 Jul 11;11(4):e149. doi: 10.1192/bjo.2025.10050 (PMC12247072; doi:10.1192/bjo.2025.10050)
Supplement: Raine et al. supplementary material 1 — Raine et al. supplementary material [file S2056472425100501sup001.docx]

## Supplementary file 1

### MEDLINE search strategy

**Ovid MEDLINE(R) ALL**

via Ovid <http://ovidsp.ovid.com/>

Date range: 1946 to October 27, 2022

Date searched: 28 October 2022

Records retrieved: 1639

The MEDLINE strategy below includes an adapted version of the DARE systematic review search filter for Ovid MEDLINE and the NICE UK search filter for Ovid MEDLINE.

Search Strategies (n.d.). About the Databases, Centre for Reviews and Dissemination. <https://www.crd.york.ac.uk/crdweb/searchstrategies.asp> Accessed: 20/10/2022

Ayiku L, Levay P, Hudson T, Craven J, Barrett E, Finnegan A and Adams R. The MEDLINE UK filter: development and validation of a geographic search filter to retrieve research about the UK from OVID MEDLINE. Health Information and Libraries Journal, 2017 34 (3): 200-216. (Publisher: Wiley. © 2017 Crown copyright. Health Information and Libraries Journal © 2017 Health Libraries Group.)

The strategy included terms to identify relevant primary studies from the UK. No geographical limits were applied to reviews.

1 exp Grief/ (9907)

2 Prolonged Grief Disorder/ (37)

3 Bereavement/ (6459)

4 (grief* or griev* or bereave* or mourn*).ti,ab,kw. (18380)

5 or/1-4 (23580)

6 systematic review/ (210522)

7 systematic$ review$.ti,ab. (268123)

8 exp meta-analysis as topic/ (25835)

9 meta-analysis/ (169629)

10 meta-analytic$.ti,ab. (9192)

11 meta-analysis.ti,ab,pt. (244080)

12 metanalysis.ti,ab. (475)

13 metaanalysis.ti,ab. (1782)

14 meta analysis.ti,ab. (213917)

15 meta-synthesis.ti,ab. (1344)

16 metasynthesis.ti,ab. (420)

17 meta synthesis.ti,ab. (1344)

18 meta-regression.ti,ab. (11716)

19 metaregression.ti,ab. (907)

20 meta regression.ti,ab. (11716)

21 (synthes$ adj3 literature).ti,ab. (5262)

22 (synthes$ adj3 evidence).ti,ab. (15785)

23 integrative review.ti,ab. (4416)

24 data synthesis.ti,ab. (13662)

25 (research synthesis or narrative synthesis).ti,ab. (6046)

26 (systematic study or systematic studies).ti,ab. (14395)

27 (systematic comparison$ or systematic overview$).ti,ab. (4270)

28 evidence based review.ti,ab. (2336)

29 comprehensive review.ti,ab. (22385)

30 critical review.ti,ab. (18704)

31 ((quantitativ* or qualitativ*) adj2 review*).ti,ab. (9016)

32 structured review.ti,ab. (989)

33 realist review.ti,ab. (536)

34 realist synthesis.ti,ab. (307)

35 ((mixed or multiple or indirect) adj treatment$ comparison$).ti,ab. (932)

36 or/6-35 (501097)

37 review.pt. (3064650)

38 medline.ab. (153541)

39 pubmed.ab. (185654)

40 cochrane.ab. (122030)

41 embase.ab. (138998)

42 cinahl.ab. (40473)

43 psyc?lit.ab. (917)

44 psyc?info.ab. (53436)

45 (literature adj3 search$).ab. (84452)

46 (database$ adj3 search$).ab. (88072)

47 (bibliographic adj3 search$).ab. (3482)

48 (electronic adj3 search$).ab. (30750)

49 (electronic adj3 database$).ab. (43088)

50 (computeri?ed adj3 search$).ab. (3851)

51 (internet adj3 search$).ab. (4083)

52 included studies.ab. (38984)

53 (inclusion adj3 studies).ab. (23226)

54 inclusion criteria.ab. (119326)

55 selection criteria.ab. (34739)

56 predefined criteria.ab. (2404)

57 predetermined criteria.ab. (1255)

58 (assess$ adj3 (quality or validity)).ab. (105460)

59 (select$ adj3 (study or studies)).ab. (85379)

60 (data adj3 extract$).ab. (89684)

61 extracted data.ab. (19343)

62 (data adj2 abstracted).ab. (6277)

63 (data adj3 abstraction).ab. (2150)

64 published intervention$.ab. (236)

65 ((study or studies) adj2 evaluat$).ab. (233907)

66 (intervention$ adj2 evaluat$).ab. (14496)

67 confidence interval$.ab. (526794)

68 heterogeneity.ab. (216078)

69 pooled.ab. (118997)

70 pooling.ab. (15549)

71 odds ratio$.ab. (343443)

72 (Jadad or coding).ab. (222910)

73 or/38-72 (1870519)

74 37 and 73 (315552)

75 review.ti. (637497)

76 75 and 73 (237222)

77 (review$ adj4 (papers or trials or studies or evidence or intervention$ or evaluation$)).ti,ab. (240198)

78 ((quantitativ* or qualitativ*) adj2 synth*).ti,ab. (8994)

79 ((scoping or rapid) adj2 (review* or stud*)).ti,ab. (23116)

80 36 or 74 or 76 or 77 or 78 or 79 (790772)

81 5 and 80 (998)

82 limit 81 to yr="2015 -Current" (656)

83 exp United Kingdom/ (386814)

84 (national health service* or nhs*).ti,ab,in. (254732)

85 (english not ((published or publication* or translat* or written or language* or speak* or literature or citation*) adj5 english)).ti,ab. (46560)

86 (gb or "g.b." or britain* or (british* not "british columbia") or uk or "u.k." or united kingdom* or (england* not "new england") or northern ireland* or northern irish* or scotland* or scottish* or ((wales or "south wales") not "new south wales") or welsh*).ti,ab,jw,in. (2362607)

87 (bath or "bath's" or ((birmingham not alabama*) or ("birmingham's" not alabama*) or bradford or "bradford's" or brighton or "brighton's" or bristol or "bristol's" or carlisle* or "carlisle's" or (cambridge not (massachusetts* or boston* or harvard*)) or ("cambridge's" not (massachusetts* or boston* or harvard*)) or (canterbury not zealand*) or ("canterbury's" not zealand*) or chelmsford or "chelmsford's" or chester or "chester's" or chichester or "chichester's" or coventry or "coventry's" or derby or "derby's" or (durham not (carolina* or nc)) or ("durham's" not (carolina* or nc)) or ely or "ely's" or exeter or "exeter's" or gloucester or "gloucester's" or hereford or "hereford's" or hull or "hull's" or lancaster or "lancaster's" or leeds* or leicester or "leicester's" or (lincoln not nebraska*) or ("lincoln's" not nebraska*) or (liverpool not (new south wales* or nsw)) or ("liverpool's" not (new south wales* or nsw)) or ((london not (ontario* or ont or toronto*)) or ("london's" not (ontario* or ont or toronto*)) or manchester or "manchester's" or (newcastle not (new south wales* or nsw)) or ("newcastle's" not (new south wales* or nsw)) or norwich or "norwich's" or nottingham or "nottingham's" or oxford or "oxford's" or peterborough or "peterborough's" or plymouth or "plymouth's" or portsmouth or "portsmouth's" or preston or "preston's" or ripon or "ripon's" or salford or "salford's" or salisbury or "salisbury's" or sheffield or "sheffield's" or southampton or "southampton's" or st albans or stoke or "stoke's" or sunderland or "sunderland's" or truro or "truro's" or wakefield or "wakefield's" or wells or westminster or "westminster's" or winchester or "winchester's" or wolverhampton or "wolverhampton's" or (worcester not (massachusetts* or boston* or harvard*)) or ("worcester's" not (massachusetts* or boston* or harvard*)) or (york not ("new york*" or ny or ontario* or ont or toronto*)) or ("york's" not ("new york*" or ny or ontario* or ont or toronto*))))).ti,ab,in. (1669388)

88 (bangor or "bangor's" or cardiff or "cardiff's" or newport or "newport's" or st asaph or "st asaph's" or st davids or swansea or "swansea's").ti,ab,in. (66879)

89 (aberdeen or "aberdeen's" or dundee or "dundee's" or edinburgh or "edinburgh's" or glasgow or "glasgow's" or inverness or (perth not australia*) or ("perth's" not australia*) or stirling or "stirling's").ti,ab,in. (246093)

90 (armagh or "armagh's" or belfast or "belfast's" or lisburn or "lisburn's" or londonderry or "londonderry's" or derry or "derry's" or newry or "newry's").ti,ab,in. (32063)

91 or/83-90 (2965673)

92 (exp africa/ or exp americas/ or exp antarctic regions/ or exp arctic regions/ or exp asia/ or exp australia/ or exp oceania/) not (exp United Kingdom/ or europe/) (3251818)

93 91 not 92 (2809273)

94 5 and 93 (3165)

95 limit 94 to yr="2015 -Current" (1296)

96 82 or 95 (1762)

97 exp animals/ not humans/ (5058782)

98 96 not 97 (1755)

99 letter.pt. (1197110)

100 editorial.pt. (624133)

101 comment.pt. (983795)

102 exp historical article/ (409053)

103 anecdotes as topic/ (4746)

104 or/99-103 (2495022)

105 98 not 104 (1646)

106 remove duplicates from 105 (1639)

**Key:**

/ or .sh. = indexing term (Medical Subject Heading: MeSH)

exp = exploded indexing term (MeSH)

* or $ =  truncation

ti,ab,kw = terms in either title, abstract, keyword fields

in = institution field

adj3 = terms within three words of each other (any order)

? = optional wild card character for zero or one letters

pt = publication type

### Websites searched

To identify relevant publications, we also searched the websites of the following organisations:

[The Center for Complicated Grief at Columbia University](https://prolongedgrief.columbia.edu/); [Cruse Bereavement Support](https://www.cruse.org.uk/);
[National Bereavement Alliance](https://nationalbereavementalliance.org.uk/all-resources/); [Sue Ryder](https://www.sueryder.org/end-of-life-care/for-healthcare-professionals/our-research/); [Marie Curie Palliative Care Research Centre](https://www.mariecurie.org.uk/research/strategy/research-centres/marie-curie-palliative-care-research-centre-cardiff); [The Caresearch Project: Palliative Care Knowledge Network](https://www.caresearch.com.au/); [Childhood Bereavement Network](https://childhoodbereavementnetwork.org.uk/); [Childhood Bereavement UK](https://www.childbereavementuk.org/); [Sands](https://www.sands.org.uk/support/bereavement-support); [The Irish Hospice Foundation](https://hospicefoundation.ie/); [MIND](https://www.mind.org.uk/); [National Bereavement Service](https://thenbs.org/); [The Good Grief Trust](https://www.thegoodgrieftrust.org/); [The Good Grief Project](https://thegoodgriefproject.co.uk/); [Marie Curie](https://www.mariecurie.org.uk/help/support/bereavement); [NHS (bereavement support)](https://www.nhs.uk/mental-health/feelings-symptoms-behaviours/feelings-and-symptoms/grief-bereavement-loss/); [Quakers Social Action](https://quakersocialaction.org.uk/sharing-our-learning/reports-publications); [Winston's Wish](https://winstonswish.org/); and [Just B](https://justb.org.uk/)

### Eligibility criteria for systematic reviews

*Study design*: Any form of systematic review. A review was considered systematic if authors (i) searched at least two sources, one of which must have been a named database, (ii) reported some search terms and clear eligibility criteria covering key review components, (iii) reported the number of references retrieved and the number of studies included, and (iv) provided a synthesis of findings. This could be a statistical synthesis in the form of a meta-analysis, or a narrative or realist synthesis.

*Population:* (i) Any bereaved individuals or population group. We did not place any restrictions on the sociodemographic characteristics; health status/co-morbidity; or place of residence of the bereaved. (ii) Key informants from any relevant organisation, such as those working in the health, social care or third sector.

*Focus (non-interventions)*: Any reviews focused on (i) identifying the frequency or risk of PGD, and/or exploring the relationship between grief-related outcomes and potential risk factors. Risk factors could be on any level including individual, social, and structural factors. This could include, but was not limited to, sociodemographic factors, poverty, discrimination, and policy-level factors such as workplace policies and COVID-19 restrictions on burial/religious practices; (ii) Experiences of bereavement, grief, use of bereavement services or interactions with other individuals or organisations following a bereavement.

*Focus (Interventions):* Reviews of interventions, which could be either, or both: (i) individual-level interventions, for example, the provision of information; psychological or drug therapies and bereavement counselling or (ii) social- or structural-level interventions aimed at addressing factors that disrupt the process of grieving or coping with bereavement, for example, changes to institutional/service organisation, delivery, policy or culture. Interventions could focus on support provided before and/or after bereavement.

*Comparators:* Any type of comparator, where applicable. For example, interventions could be compared with usual treatment, an alternate treatment, waiting-list control, no treatment, or a before-and-after intervention assessment.

*Outcomes (non-intervention reviews)*: Reviews that reported findings related to any of the following: prevalence, incidence or relative risk/odds of PGD; factors that assist or impair the bereavement/grieving process; enablers and barriers to the use of bereavement support; post bereavement needs; views about using support services or other sources of support, e.g., family, friends; staff views about bereavement service provision and the needs of the bereaved. Factors could be at any level, e.g., personal/interpersonal/cultural/social/economic/institutional.

*Outcomes (intervention reviews*): Reviews of interventions that reported any outcome related to the effectiveness, cost-effectiveness, or safety of interventions or their impact on health inequities. Studies could also report outcomes related to the implementation of interventions and factors that influence effectiveness. For the purposes of our review, effectiveness was conceptualised broadly to include findings from qualitative studies relating to the perceived impact and benefits of interventions.

### Supplementary search (November 2024)

A supplementary search was conducted by an information specialist (HF) on 25th November 2024. We sought to identify any reviews published since the original search in October 2022 that addressed the key evidence gaps identified from our evidence map. The supplementary search was run on six databases likely to contain reviews: MEDLINE; Embase; PsycINFO; CINAHL Complete; Cochrane Database of Systematic Reviews; and Epistemonikos. Date limits of 2022-Current were used in all search strategies. The results from the six databases were deduplicated against each other before being merged with the results of the original EndNote Library and deduplicated for a second time, identifying 542 unique records.

### MEDLINE search strategy

**Ovid MEDLINE(R) ALL**

via Ovid <http://ovidsp.ovid.com/>

Date range searched: <1946 to November 22, 2024>

Date searched: 25 November 2024

Records retrieved: 392

The MEDLINE strategy below includes an adapted version of the DARE systematic review search filter for Ovid Medline and the NICE UK search filter for Ovid Medline.

Search Strategies (n.d.). About the Databases, Centre for Reviews and Dissemination. <https://www.crd.york.ac.uk/crdweb/searchstrategies.asp>

1 exp Grief/ (10645)

2 Prolonged Grief Disorder/ (99)

3 Bereavement/ (7079)

4 (grief* or griev* or bereave* or mourn*).ti,ab,kw. (20947)

5 or/1-4 (26159)

6 systematic review/ (280736)

7 systematic$ review$.ti,ab. (362045)

8 exp meta-analysis as topic/ (31419)

9 meta-analysis/ (212418)

10 meta-analytic$.ti,ab. (10904)

11 meta-analysis.ti,ab,pt. (310346)

12 metanalysis.ti,ab. (736)

13 metaanalysis.ti,ab. (1934)

14 meta analysis.ti,ab. (278796)

15 meta-synthesis.ti,ab. (1884)

16 metasynthesis.ti,ab. (491)

17 meta synthesis.ti,ab. (1884)

18 meta-regression.ti,ab. (15554)

19 metaregression.ti,ab. (1019)

20 meta regression.ti,ab. (15554)

21 (synthes$ adj3 literature).ti,ab. (7223)

22 (synthes$ adj3 evidence).ti,ab. (21310)

23 integrative review.ti,ab. (5555)

24 data synthesis.ti,ab. (15719)

25 (research synthesis or narrative synthesis).ti,ab. (9005)

26 (systematic study or systematic studies).ti,ab. (16278)

27 (systematic comparison$ or systematic overview$).ti,ab. (5164)

28 evidence based review.ti,ab. (2538)

29 comprehensive review.ti,ab. (35408)

30 critical review.ti,ab. (21022)

31 ((quantitativ* or qualitativ*) adj2 review*).ti,ab. (11554)

32 structured review.ti,ab. (1100)

33 realist review.ti,ab. (750)

34 realist synthesis.ti,ab. (427)

35 ((mixed or multiple or indirect) adj treatment$ comparison$).ti,ab. (1070)

36 or/6-35 (641072)

37 review.pt. (3415935)

38 medline.ab. (187150)

39 pubmed.ab. (257215)

40 cochrane.ab. (160291)

41 embase.ab. (186209)

42 cinahl.ab. (53681)

43 psyc?lit.ab. (919)

44 psyc?info.ab. (69235)

45 (literature adj3 search$).ab. (107523)

46 (database$ adj3 search$).ab. (114866)

47 (bibliographic adj3 search$).ab. (4272)

48 (electronic adj3 search$).ab. (38223)

49 (electronic adj3 database$).ab. (55161)

50 (computeri?ed adj3 search$).ab. (4158)

51 (internet adj3 search$).ab. (4682)

52 included studies.ab. (55035)

53 (inclusion adj3 studies).ab. (29936)

54 inclusion criteria.ab. (151580)

55 selection criteria.ab. (38880)

56 predefined criteria.ab. (2886)

57 predetermined criteria.ab. (1446)

58 (assess$ adj3 (quality or validity)).ab. (131286)

59 (select$ adj3 (study or studies)).ab. (102798)

60 (data adj3 extract$).ab. (115739)

61 extracted data.ab. (23840)

62 (data adj2 abstracted).ab. (6970)

63 (data adj3 abstraction).ab. (2534)

64 published intervention$.ab. (271)

65 ((study or studies) adj2 evaluat$).ab. (278819)

66 (intervention$ adj2 evaluat$).ab. (17518)

67 confidence interval$.ab. (623762)

68 heterogeneity.ab. (267298)

69 pooled.ab. (144590)

70 pooling.ab. (18380)

71 odds ratio$.ab. (407050)

72 (Jadad or coding).ab. (251389)

73 or/37-72 (5266888)

74 36 and 73 (514892)

75 review.ti. (802503)

76 75 and 73 (616345)

77 (review$ adj4 (papers or trials or studies or evidence or intervention$ or evaluation$)).ti,ab. (287863)

78 ((quantitativ* or qualitativ*) adj2 synth*).ti,ab. (12360)

79 ((scoping or rapid) adj2 (review* or stud*)).ti,ab. (40326)

80 36 or 74 or 76 or 77 or 78 or 79 (1079559)

81 5 and 80 (1282)

82 limit 81 to yr="2022 -Current" (403)

83 exp animals/ not humans/ (5279972)

84 82 not 83 (401)

85 letter.pt. (1280148)

86 editorial.pt. (713696)

87 comment.pt. (1045188)

88 exp historical article/ (414321)

89 anecdotes as topic/ (4747)

90 or/85-89 (2691166)

91 84 not 90 (399)

92 remove duplicates from 91 (392)

**Key:**

/ or .sh. = indexing term (Medical Subject Heading: MeSH)

exp = exploded indexing term (MeSH)

* or $ =  truncation

ti,ab,kw = terms in either title, abstract, keyword fields

adj3 = terms within three words of each other (any order)

? = optional wild card character for zero or one letters

pt = publication type
