# Supplementary material for: Grief, bereavement and prolonged grief disorder: scoping and mapping the evidence
Source: BJPsych Open. 2025 Jul 11;11(4):e149. doi: 10.1192/bjo.2025.10050 (PMC12247072; doi:10.1192/bjo.2025.10050)
Supplement: Raine et al. supplementary material 2 — Raine et al. supplementary material [file S2056472425100501sup002.docx]

## Supplementary file 2: Key characteristics of included reviews (n=212)

| **First author (year)** | **Review aim** | **Review type**  **Number of included studies** | **Focus** | **Characteristics of the bereaved**  **Restrictions on inclusion**  **Nature of death** |
| --- | --- | --- | --- | --- |
| Aehlig (2021) | To review the current knowledge about associations between attachment styles and prolonged grief in bereaved adults | Systematic review n=25 | Risk factors for PGD | Family or friends Adults  No specific cause/type of death |
| Ainscough (2022) | To assess the effectiveness of bereavement support interventions for parents of an infant or a child who has died from a medical condition or in unforeseen circumstances | Systematic review n=8 (9 articles) | Intervention effectiveness  Intervention type: Non-specific | Parents  No specific cause/type of death |
| Albuquerque (2016) | To present the current knowledge on the effects of the death of a child on the marital relationship (and associated variables), and on the effect of the marital relationship on parents’ individual adjustment to the loss | Systematic review n=24 | General grief reactions | Parents  No specific cause/type of death |
| Alves-Costa (2021) | To review the psychological interventions available for individuals bereaved by homicide and report their effectiveness | Systematic review n=7 | Intervention effectiveness  Intervention type: Psychological | Family or friends  Violent or unnatural death - Homicide |
| Anderson (2022) | To explore factors that influence the process of adjustment following the death of a partner at a younger than expected age | Systematic review n=13 | Grief and bereavement experiences | Spouse or partner  Adults 50 years or younger  No specific cause/type of death |
| Andriessen (2016) | To disentangle pre- and post-loss features that affect response to grief in adolescents bereaved by suicide | Systematic review n=58 | General grief reactions  Grief and bereavement experiences | Non-specific relationship to the deceased  Children or young people  Suicide |
| Andriessen (2019) | To assess the evidence of effectiveness of interventions for people bereaved by suicide | Systematic review n=11 (12 articles) | Intervention effectiveness  Intervention type: Non-specific | Non-specific relationship to the deceased  Suicide |
| Andriessen (2019) | To investigate what is known of the effects of postvention service delivery models and the components that may contribute to the effectiveness | Systematic review n=8 (plus 12 guidelines) | Intervention effectiveness  Intervention type: Structured/formal support | Non-specific relationship to the deceased  Suicide |
| Andriessen (2020) | To assess what is known about the grief and mental health of people bereaved by euthanasia or physician-assisted suicide | Systematic review n=8 (10 articles) | Risk factors for PGD  Grief and bereavement experiences | Non-specific relationship to the deceased  Assisted suicide/euthanasia |
| Arruda (2017) | To examine and synthesise the evidence on grief and bereavement in Alzheimer’s disease and related dementias caregivers | Systematic review n=19 (including 1 SR) | General grief reactions  Grief and bereavement experiences  Intervention effectiveness   Intervention type: Non-specific | Informal/family carers  Health condition or terminal illness (Alzheimer's disease and related dementias) |
| Asgari (2022) | To synthesise the results of studies about grief interventions and their effectiveness | Review of reviews n=16 | Intervention effectiveness  Intervention type: Non-specific | Non-specific relationship to the deceased  No specific cause/type of death |
| Baffour-Awuah (2020)^a^ | To explore and summarise the existing literature about the experiences of perinatal loss among couples in sub-Saharan Africa, and to identify the relevant support health professionals and the community give to improve the well-being of parents experiencing perinatal loss | Systematic review n=8 | Grief and bereavement experiences | Parents  Sub-Saharan Africa  Perinatal loss |
| Barnes (2020) | To synthesise the grief experiences of health professionals associated with paediatric death in healthcare settings | Systematic review n=12 (13 articles) | Grief and bereavement experiences | Professionals - healthcare  No specific cause/type of death |
| Bartone (2019) | To assess the evidence regarding benefits of peer support services for bereaved survivors of sudden or unexpected death | Systematic review n=32 | Intervention effectiveness  Intervention type: Peer support | Family member, close friend, or co-worker  Sudden or unexpected death |
| Bergman (2017) | To systematically review studies about effective support interventions for parentally bereaved children and to identify gaps in the research | Systematic review n=17 | Intervention effectiveness  Intervention type: Non-specific | Child or their remaining caregiver  Children and young people  No specific cause/type of death |
| Berry (2021) | To identify and synthesise common, experiential themes from qualitative studies of parents who experienced perinatal loss | Systematic review n=5 | Grief and bereavement experiences | Parents  Perinatal loss |
| Bindley (2019) | To summarise published research on experiences of social and structural inequities in the context of bereavement due to life-limiting illness | Systematic review n=15 | Grief and bereavement experiences | Non-specific relationship to the deceased  Adults; high-income countries  Health condition or terminal illness (expected death of an adult) |
| Bjelland (2022) | To identify factors that relieve or contribute to distress for deceased organ donor families in the time since the decision to donate | Systematic review n=17 | Risk factors for PGD  General grief reactions  Grief and bereavement experiences | Family or friends  No specific cause/type of death |
| Bloomer (2022) | To synthesise research evidence on the experience of the nurse after the death of a patient in adult critical care | Systematic review n=35 (36 articles) | Grief and bereavement experiences | Professionals - Nurses  No specific cause of death - specific setting (ICU/critical/care) |
| Boven (2022) | To identify the role hospitals take in providing bereavement care after an in-hospital adult death | Systematic review n=47 | Grief and bereavement experiences | Non-specific relationship to the deceased  No specific cause of death - specific setting (hospital death) |
| Brekelmans (2022) | To give an overview of different bereavement support strategies and possible different effects on anxiety, depression, post-traumatic stress disorder, and complicated grief | Systematic review n=7 | Intervention effectiveness  Intervention type: Non-specific (hospital settings) | Family or friends  No specific cause of death - specific setting (ICU/critical care) |
| Bristowe (2016) | To identify and appraise the evidence of the bereavement experiences of lesbian, gay, bisexual and/or trans people who have lost a partner | Systematic review n=13 (23 articles) | Grief and bereavement experiences | Spouse or partner LGBT+  No specific cause/type of death |
| Brown-Brundage (2021) | To document the grief experiences of long-term care staff and benefits of end-of-life planning | Systematic review n=19 | General grief reactions  Grief and bereavement experiences | Professionals: long-term care staff  No specific cause of death - specific setting (long-term care) |
| Burden (2016) | To systematically review, evaluate and summarise the current evidence regarding the psychosocial impact of stillbirth to parents and their families | Systematic review n=144 | Grief and bereavement experiences | Parents with family (grandparents, siblings, or future children, including a surviving multiple)   Perinatal loss |
| Burrell (2022) | To synthesise evidence on the impact of funeral practices on bereaved friends and relatives’ mental health and experience of bereavement | Systematic review n=17 | Risk factors for PGD  General grief reactions | Family or friends   No specific cause/type of death |
| Butler (2015) | To review, critique and synthesise current research studies that examine parental perceptions of healthcare provider actions during and after the death of a child | Systematic review n=15 | Grief and bereavement experiences | Parents  No specific cause/type of death |
| Butler (2015) | To review and synthesise the best available evidence exploring the family experience of the death of their child in the paediatric ICU | Systematic review n=15 | Grief and bereavement experiences | Parents with family (siblings, grandparents, or legal guardians)  No specific cause of death - specific setting (paediatric ICU) |
| Byeon (2020) | To review systematically the effects of grief-focused interventions, applied to dementia caregivers, on emotional burden including sadness | Systematic review n=5 | Intervention effectiveness  Intervention type: Non-specific | Informal/family carers  Health condition or terminal illness (dementia) |
| Causer (2019) | To address the question "what are the features of the experiences of workers in health, education or social care roles following the death by suicide of a client, patient, student or service user?" | Systematic review n=11 (12 articles) | Grief and bereavement experiences | Professionals: Health, education or social care  Suicide |
| Causer (2022) | To explore the impact of colleague suicide on surviving co-workers and review postvention guidance for workplaces | Systematic review n=17 | Grief and bereavement experiences | Work colleague  Suicide |
| Charrois | To evaluate the effectiveness of psychotherapeutic interventions and compare the content and delivery methods of interventions associated with greatest reductions in psychological distress in women after perinatal loss | Systematic review n=13 | Intervention effectiveness  Intervention type: Psychological | Parents (mothers) Adults; females  Perinatal loss |
| Cheer (2016) | To deepen understanding of how Asia-Paciﬁc women experience the phenomenon of still-birth | Systematic review n=6 (7 articles) | Grief and bereavement experiences | Parents (mothers) Females; Asia-Pacific  Perinatal loss |
| Chen (2018) | To systematically review and synthesise the experience of bereavement after patients' deaths, and in particular, its core process, in professional caregivers' own descriptions | Systematic review n=23 | Grief and bereavement experiences | Professionals: Healthcare   No specific cause/type of death |
| Chen (2018) | To identify interventions for bereaved preschool-age children and examine their effectiveness | Systematic review n=17 | Intervention effectiveness  Intervention type: Non-specific | Child  Children and young people (3-5 years)  No specific cause/type of death |
| Chen (2019) | A scoping review of quantitative research that examines (a) how researchers define and measure professional caregivers’ bereavement after patients’ deaths; and (b) what the findings are | Systematic review n=12 | General grief reactions  Grief and bereavement experiences | Professionals - Healthcare  No specific cause/type of death |
| Clabburn (2021) | To investigate how bereaved young people continue bonds with deceased family members | Systematic review  n=20 | Grief and bereavement experiences | Child Children and young people (24 years or younger)  No specific cause/type of death |
| Coelho (2017) | To synthesise recent research to develop further knowledge about the family experience of anticipatory grief during a patient’s end of life | Systematic review n=29 | Grief and bereavement experiences | Family or friends Adults   Health condition or terminal illness (advanced disease/end-of-life) |
| Coffey (2016) | To gain an understanding of what parents perceived as good practice in care during and after stillbirth | Systematic review n=8 | Grief and bereavement experiences | Parents  High-income countries with maternity services comparable to the UK  Perinatal loss |
| Connolly (2015) | To conduct a systematic review on the effects of homicide on surviving family members | Systematic review n=40 | Grief and bereavement experiences | Family or friends  Violent or unnatural death (homicide) |
| Crawley (2022) | To report the prevalence of pre-death and post-death grief and to synthesise associated factors and the relationship between pre-death factors and post-death grief and services used to manage grief | Systematic review n=55 | Extent of PGD  Risk factors for PGD  General grief reactions | Informal/family carers Adults   Health condition or terminal illness (dementia) |
| Crispus (2015) | To summarise and critically evaluate the evidence informing the provision of standard care practices and psychosocial interventions following stillbirth | Systematic review n=20 | Intervention effectiveness  Intervention type: Psychosocial | Parents  Perinatal loss |
| D'Alton (2022) | To identify potential risk and protective factors for maladaptive coping following sibling bereavement and the inﬂuence of these factors on adjustment to loss | Systematic review n=25 | General grief reactions  Grief and bereavement experiences | Sibling Children and young people  No specific cause/type of death |
| Davidow (2022) | To examine the interventions and outcomes that have been studied for late-life spousal bereavement | Systematic review n=22 | Intervention effectiveness  Intervention type: Non-specific | Spouse or partner Adults (average age over 50 years)  No specific cause/type of death |
| de Andrade Alvarenga (2021) | To synthesise qualitative evidence from primary studies to better understand the experience of the spirituality of parents and its relationship to adapting following stillbirth | Systematic review n=21 | Grief and bereavement experiences | Parents  Perinatal loss |
| de Lopez (2020) | To evaluate the eﬀect of grief interventions for children and adolescents who have lost a parent or sibling | Systematic review n=8 | Intervention effectiveness  Intervention type: Non-specific | Child/sibling Children and young people  No specific cause/type of death |
| De Rosbo-Davies (2022) | To synthesise peer-reviewed literature on daughters' experiences of maternal bereavement | Systematic review n=21 | Grief and bereavement experiences | Daughter - death of mother (at any age up to 50 years) Females  No specific cause/type of death |
| Delalibera (2015) | To investigate the effect of family dynamics on adult family members’ grieving process | Systematic review n=15 | Risk factors for PGD  General grief reactions | Family or friends Adults  No specific cause/type of death |
| Dias (2019) | To describe the interventions for bereaved parents and evaluate intervention effectiveness | Systematic review n=9 | Intervention effectiveness  Intervention type: Non-specific | Parents  Health condition or terminal illness (acute or chronic illness) |
| Djelantik (2020) | To estimate a pooled prevalence of prolonged grief disorder in individuals bereaved due to unnatural causes | Systematic review n=25 | Extent of PGD  Risk factors for PGD | Non-specific relationship to the deceased  Adults  Violent or unnatural death (unnatural loss) |
| Dolan (2022) | To present the effectiveness of cognitive behavioural therapy and mindfulness-based interventions for perinatal grief, to report patient experiences of the interventions, and to determine which intervention can be more effective in managing symptoms of complicated perinatal grief | Systematic review n=8 | Intervention effectiveness  Intervention type: CBT and mindfulness therapy | Parents  Perinatal loss |
| Donovan (2015) | To identify services offered to bereaved families in perinatal, neonatal, and paediatric hospital settings and summarise the psychosocial impact of these services and published recommendations for best practice hospital-based bereavement care | Systematic review n=34 | Grief and bereavement experiences  Intervention effectiveness  Intervention type: Non-specific (hospital settings) | Parents with family (siblings or grandparents for interventions)  No specific cause of death - specific setting (hospital settings) |
| Dorman (2022) | To find and describe literature relating to family experiences in paediatric hospice palliative care throughout the end-of-life care journey including grief and bereavement | Systematic review n=9 (10 articles) | Grief and bereavement experiences | Family or friends   Health condition or terminal illness (paediatric hospices) |
| Duncan (2015) | To assess evidence on the prevalence of self-blame, guilt, and shame in bereaved parents, as well as the relationships between self-blame, guilt, and shame and any measures of psychological adaptation after loss | Systematic review n=18 | General grief reactions | Parents Adults  No specific cause/type of death |
| Duncan (2020) | To identify approaches used to support children who are grieving, and to explore implications for teachers | Systematic review n=15 | Grief and bereavement experiences | Child  Children and young people (3 to 18 years)  No specific cause/type of death |
| Dutta (2019) | To understand the lived experience of parents who have lost their child to a chronic life-limiting condition | Systematic review n=25 | Grief and bereavement experiences | Parents  Adults   Health condition or terminal illness (chronic life-limiting illness) |
| Eddinger (2021) | To produce an overview of treatments used to concurrently reduce symptoms of posttraumatic stress disorder and prolonged grief disorder | Systematic review n=14 | Intervention effectiveness  Intervention type: Non-specific | Non-specific relationship to the deceased  Adults  No specific cause/type of death |
| Efstathiou (2019) | To investigate the state of ICU bereavement support globally, and the availability and effectiveness of bereavement support interventions | Systematic review n=14 | Intervention effectiveness  Intervention type:  Non-specific (hospital settings) | Family or friends   No specific cause of death - specific setting (Adult ICU) |
| Eisma (2021) | To identify quantitative research examining relationships between emotion regulation and complicated grief | Systematic review n=64 | Risk factors for PGD | Family or friends   No specific cause/type of death |
| Ellis (2016) | To analyse the available evidence on parents’ views on the experience of going through a stillbirth, or key healthcare workers experiences of caring for couples dealing with a stillbirth | Systematic review n=52 | Grief and bereavement experiences | Parents or healthcare workers High-income countries (Europe, North America, Australia and South Africa)  Perinatal loss |
| Endo (2015) | To evaluate the efficacy of interventions for bereaved parents and siblings following a child's death | Systematic review n=8 (9 articles) | Intervention effectiveness  Intervention type: Non-specific | Parents with family (siblings)  No specific cause/type of death |
| Enez (2017) | To investigate which psychotherapy-based intervention are designed for the treatment of complicated grief and to assess the effectiveness of these interventions | Systematic review n=21 | Intervention effectiveness  Intervention type: Psychological | Non-specific relationship to the deceased Adults   No specific cause/type of death |
| Evans (2020) | To evaluate the impact of public stigma on bereavement of suicide survivors | Systematic review n=11 | General grief reactions  Grief and bereavement experiences | Family or friends Adults  Suicide |
| Falzarano (2022) | To synthesise the literature documenting the bereavement experiences of the Latino/a community, evaluate the strength of the evidence, and provide recommendations for research | Systematic review n=26 (including 1 SR; 2 commentaries and 5 narrative reviews) | Grief and bereavement experiences | Non-specific relationship to the deceased  Adults; Latino or Latina  No specific cause/type of death |
| Fee (2021) | To synthesise qualitative research evidence reporting adults' experiences of pre-loss grief within cancer care | Systematic review n=13 | Grief and bereavement experiences | Non-specific relationship to the deceased  Adults  Health condition or terminal illness (cancer) |
| Fernandez-Basanta (2020) | To synthesise research findings regarding the coping experiences of parents following perinatal loss | Systematic review n=14 | Grief and bereavement experiences | Parents  Perinatal loss |
| Fernandez-Ferez (2021) | To determine the efficacy of nursing interventions to facilitate the process of grief as a result of perinatal death | Systematic review n=4 | Intervention effectiveness  Intervention type: Non-specific (nursing) | Parents (mothers) Females  Perinatal loss |
| Fernandez-Fernandez (2022) | To find out whether there is a relationship between the experience of grief facing the loss of a loved one and the development of resilience | Systematic review  n=24 | General grief reactions | Non-specific relationship to the deceased  No specific cause/type of death |
| Finlayson-Short (2020) | To systematically review the quality of evidence regarding the effectiveness of supports for people affected by suicide | Systematic review n=15 (1 not bereaved) | Intervention effectiveness  Intervention type: Community-based | Non-specific relationship to the deceased  Suicide |
| Fiore (2021) | To investigate whether the Dual Process Model of Coping with Bereavement (DPM) accurately represents the bereavement experience, and whether DPM-based interventions are more effective than traditional grief therapy | Systematic review n=22 | General grief reactions  Grief and bereavement experiences  Intervention effectiveness  Intervention type: based on the dual process model of coping | Family or friends  No specific cause/type of death |
| Flach (2022) | To identify the factors associated with the development and prevention of complicated grief in women who have lost a baby | Systematic review n=23 | Risk factors for PGD | Parents (mothers) Adults; females  Perinatal loss |
| Galazzi (2022) | To investigate the connection between intensive care unit diaries and the grieving process experienced by family members of adult patients deceased in the intensive care unit | Systematic review n=6 | Intervention effectiveness   Intervention type: ICU diaries | Parents with family (spouses, daughters, sons, and close friends)  No specific cause of death: specific setting (ICU/critical care) |
| Gamondi (2019) | To systematically review family experiences of assisted dying | Systematic review n=19 | Grief and bereavement experiences | Informal/family carers  Assisted suicide/euthanasia |
| Garcini (2021) | To systematically review the methodology and findings of scientific studies and reviews of bereavement among widowed Latinos in the USA | Systematic review n=19 (including 6 narrative reviews) | Grief and bereavement experiences  General grief reactions | Spouse or partner Latinos; USA  No specific cause/type of death |
| Goodall (2022) | To analysing the research on continuing bonds in individuals bereaved by suicide. It aims to characterise aspects of the continuing bond including how they manifest, and whether those bereaved experience them as positive or negative | Systematic review n=15 | Grief and bereavement experiences | Non-specific relationship to the deceased  Suicide |
| Grijo (2021) | To explore the outcomes of dignity therapy in palliative care patients' family members | Systematic review n=8 | Intervention effectiveness/  Implementation  Intervention type: Dignity therapy | Family or friends Adults  Health condition or terminal illness (end of life) |
| Grose (2018) | To synthesise findings on the effect of perceived caregiver burden on post-bereavement mental health in bereaved caregivers of cancer patients | Systematic review n=14 (20 articles) | Extent of PGD  Risk factors for PGD | Informal/family carers Adults  Health condition or terminal illness (cancer) |
| Hai (2018) | To investigate the spirituality/religiosity’s relationship with bereavement among college students | Systematic review n=8 | General grief reactions | Non-specific relationship to the deceased  Adult college students (17 to 34 years); USA  No specific cause/type of death |
| Hanschmidt (2016) | To provide a critical overview on the current state of research on suicide survivor stigma and to investigate the influence of stigma on grief trajectories and survivors’ well-being | Systematic review n=25 | Risk factors for PGD  General grief reactions | Non-specific relationship to the deceased  Suicide |
| Harrop (2020) | To review the quantitative and qualitative evidence on the effectiveness and impact of interventions and services providing support for adults bereaved through advanced illness | Systematic review n=31 | Intervention effectiveness  Intervention type: Non-specific | Non-specific relationship to the deceased  Adults  Health condition or terminal illness (advanced illness) |
| Harrop (2020) | To synthesise the evidence regarding system-level responses to mass bereavement events | Systematic review n=6 | Intervention effectiveness/ implementation  Intervention type: systems-based approaches | Non-specific relationship to the deceased  Mass events (natural or human-made disasters as well as pandemics) |
| Hay (2022) | To identify how grief affects students in higher education and the types of support they seek and/or ﬁnd beneﬁcial | Systematic review n=30 | Extent of PGD  Risk factors for PGD  General grief reactions  Grief and bereavement experiences | Non-specific relationship to the deceased  Adults (students in higher education)   No specific cause/type of death |
| Haylett (2021) | To determine the current state of science regarding bereaved parenting | Systematic review n=20 | General grief reactions  Grief and bereavement experiences | Parents  No specific cause/type of death |
| Heazell (2016) | To establish the effect of stillbirth on parents, families, health-care providers, and societies worldwide | Systematic review n= 42 or 43 on interventions; 144 on experiences | Grief and bereavement experiences  Intervention effectiveness  Intervention type: Non-specific | Parents with family   Perinatal loss |
| Heeke (2019) | To identify the correlates of PGD in adults exposed to violent loss | Systematic review n=37 | Risk factors for PGD | Family or friends Adults  Violent or unnatural death |
| Hennegan (2015) | To collate and critically appraise extant evidence for the impact of contact with the stillborn infant on parental mental health, well-being, and satisfaction | Systematic review n=11 (18 articles) | Intervention effectiveness  Intervention type: Parental contact with stillborn baby | Parents  Perinatal loss |
| Hewison (2019) | To summarise the evidence concerning the provision and effectiveness of bereavement support in the UK; to examine the role of informal support in bereavement; and to determine the effect of unsupported bereavement in the UK to identify gaps in service provision and areas of need for bereavement services | Systematic review n=23 | Grief and bereavement experiences  Intervention effectiveness  Intervention type: non-specific | Non-specific relationship to the deceased  Adults; UK  No specific cause/type of death |
| Higgins (2022) | To examine peer-led interventions for people bereaved by suicide | Systematic review n=10 | Intervention effectiveness  Intervention type: Peer support | Non-specific relationship to the deceased  Suicide |
| Ho (2022) | To identify, evaluate, and summarise the findings of all relevant individual studies regarding spontaneous miscarriage psychological treatment and patient experiences in various clinical settings in the USA | Systematic review n=6 | Grief and bereavement experiences  Intervention effectiveness  Intervention type: Non-specific | Parents (mothers) Adults; females; USA  Perinatal loss |
| Hoffmann (2018) | To integrate findings on psychosocial outcomes after parental or sibling cancer bereavement | Systematic review n=24 | General grief reactions | Child/sibling Children and young people   Health condition or terminal illness (cancer) |
| Holm (2019) | To examine factors that influence the health of older widows and widowers | Systematic review n=12 | Extent of PGD  General grief reactions  Grief and bereavement experiences | Spouse or partner Adults aged 60 years or older  No specific cause/type of death |
| Holtslander (2017) | To conduct a qualitative meta-summary to explore the experiences of bereaved informal/family carers of people who received palliative care services, regardless of their underlying disease | Systematic review n=47 | Grief and bereavement experiences | Informal/family carers  Health condition or terminal illness (end of life) |
| Huberty (2017) | To identify and evaluate intervention studies that target mental and/or physical health outcomes in women who have experienced stillbirth | Systematic review n=2 | Intervention effectiveness  Intervention type: Non-specific | Parents (mothers)  Females   Perinatal loss |
| Ing (2022) | To assess the availability and efficacy of interventions open to adolescents and young adults bereaved by a parent’s or sibling’s cancer | Systematic review n=40 | Intervention effectiveness  Intervention type: Psychosocial | Child/sibling Children and young people (15 to 25 years)  Health condition or terminal illness (cancer) |
| Ito (2022) | To explore previous findings about families’ experiences of grief and bereavement in the emergency department to clarify and understand their experience | Systematic review n=20 | Grief and bereavement experiences | Family or friends   No specific cause of death - specific setting (emergency departments) |
| Ito (2023) | To identify risk factors for post-intensive care syndrome-family and determine the effect size of individual risk factors | Systematic review n=17 (2 on PGD) | Risk factors for PGD | Family or friends  No specific cause of death - specific setting  (ICU deaths) |
| Jackson (2019)^a^ | To explore the experiences of pre- and post-bereaved carers, and the information that they receive in the acute hospital setting | Systematic review n=10 | Grief and bereavement experiences | Informal/family carers  No specific cause of death - specific setting (acute hospital death) |
| Jessop (2022) | To systematically review the health impact of expected parental death on adolescent and young adult children and provide a basis for further research and clinical practice | Systematic review n=10 | Extent of PGD  Risk factors for PGD  General grief reactions | Child Children and young people (15 to 25 years)  Health condition or terminal illness |
| Jiao (2021) | To identify what is already known about parent–child relationships in widowed families | Systematic review n=36 | Risk factors for PGD  General grief reactions | Child and spouse (surviving parent)  No specific cause/type of death |
| Johannsen (2019) | To evaluate the efficacy of psychological interventions for grief in bereaved adults and explore the possible moderating influence of various study characteristics | Systematic review n=31 RCTs | Intervention effectiveness  Intervention type: Psychological | Non-specific relationship to the deceased Adults   No specific cause/type of death |
| Jones (2019) | To report on research conducted on men's experiences of grief and loss following stillbirth and neonatal death in high-income, Western countries | Systematic review (including 6 SRs) n=27 | Grief and bereavement experiences | Parents (fathers) Males; high-income countries (UK, Europe, North America and Australia)  Perinatal loss |
| Jones (2022) | To investigate the effectiveness of Acceptance and Commitment Therapy for managing grief experienced by bereaved spouses or partners of adults who had received palliative care | Systematic review n=2 | Intervention effectiveness  Intervention type: Acceptance and Commitment Therapy | Spouse or partner Adults   Health condition or terminal illness (end of life) |
| Journot-Reverbel (2017) | To assess psychosocial interventions specifically targeting children and adolescents bereaved by suicide | Systematic review n=2 | Intervention effectiveness  Intervention type: Psychosocial (support groups) | Child/sibling Children and young people  Suicide |
| Kabatchnick (2016)^a^ | To explore the sequelae and support of surviving siblings of completed suicide victims in the United States | Systematic review n= not reported (book chapter) | Grief and bereavement experiences | Sibling Children and young people; USA  Suicide |
| Kakarala (2020) | To evaluate the extent to which neurological reward pathways are associated with prolonged grief disorder | Systematic review n=24 | Risk factors for PGD | Non-specific relationship to the deceased  No specific cause/type of death |
| Kaspersen (2022) | To investigate the follow-up and support offered by health services, peer support services, and other resources available (e.g., internet-based resources) for families bereaved by suicide | Systematic review n=63 | Grief and bereavement experiences   Intervention effectiveness  Intervention type: Non-specific | Defined parents with family (parents, spouse/partner, siblings, and children)  High-income countries  Suicide |
| Kenny (2020) | To examine the social support needs of parents bereaved from childhood cancer | Systematic review n=11 | Grief and bereavement experiences | Parents  Health condition or terminal illness (cancer) |
| Killikelly (2018) | To review recent studies of grief in refugees and post-conﬂict survivors in terms of the type of approach to cultural adaptation (etic or emic), the unique culturally relevant symptoms of grief revealed and the rates of disordered grief identiﬁed across the diﬀerent etic and emic approaches | Systematic review n=24 | Extent of PGD | Non-specific relationship to the deceased  Adults; refugees/migrants/asylum seekers/people living in a (post-) conflict zone  No specific cause/type of death |
| King (2022) | To explore the effects of early parental death on bereaved children's mental health | Systematic review n=8 | Risk factors for PGD  General grief reactions | Child Children and young people  No specific cause/type of death |
| Kingdon (2015) | To identify healthcare worker practices that parents’ value. The research question was how does the approach of healthcare professionals to seeing and holding the baby following stillbirth impact parents’ views and experiences? | Systematic review n=12 | Grief and bereavement experiences | Parents  Perinatal loss |
| Kochen (2020) | To provide an overview of well-defined bereavement interventions performed by regular health care professionals, and aimed at supporting parents in coping with loss, during both the end of their child’s life and after their child’s death, including effectiveness | Systematic review n=21 | Intervention effectiveness  Intervention type: Non-specific (health care professionals) | Parents  No specific cause/type of death |
| Kokou-Kpolou (2020) | To review the prevalence rates of prolonged grief disorder, its comorbidities, and associated risk factors among adult refugees | Systematic review n=12 | Extent of PGD  Risk factors for PGD | Non-specific relationship to the deceased  Adults; refugees, immigrants or asylum seekers  No specific cause/type of death |
| Komischke-Konnerup (2021) | To provide pooled prevalence estimates of the co-occurrence of prolonged grief disorder and other types of complicated grief reactions (depression, anxiety, and posttraumatic stress) | Systematic review n=23 | Extent of PGD  Risk factors for PGD | Non-specific relationship to the deceased  Adults  No specific cause/type of death |
| Kuforiji (2022) | To develop deeper understanding of women's experience of care and support following perinatal death in high-burden settings | Systematic review n=8 | Grief and bereavement experiences | Parents (mothers)  Females; ‘high burden' countries - with high perinatal death rates (Sub-Saharan Africa and South Asia)  Perinatal loss |
| Kuo (2017) | To synthesise the concepts of distinct depressive-symptom trajectories in previous studies by establishing a measurable standard and to estimate the prevalence of each re-categorised trajectory for family members grieving after the death of chronically ill patients | Systematic review n=6 | Extent of PGD | Family or friends Adults  Health condition or terminal illness (chronic disease) |
| Kustanti (2021) | To summarise and synthesise the effectiveness of bereavement support for adult informal/family carers in palliative care | Systematic review n=19 RCTs | Intervention effectiveness  Intervention type: Bereavement support (group or individual support) | Informal/family carers Adults  Health condition or terminal illness (end of life) |
| Kustanti (2022) | To determine the prevalence of grief disorders among families of patients with cancer | Systematic review n=19 | Extent of PGD  Risk factors for PGD | Family or friends Adults  Health condition or terminal illness (cancer) |
| Lalande (2022) | To better understand the experience of bereaved parents using support group services | Systematic review n=16 | Grief and bereavement experiences | Parents  Perinatal loss |
| Lancel (2020) | To establish the state of knowledge about the reciprocal relationship between grief and sleep difficulties or disorders | Systematic review n=85 (8 on complicated grief) | Risk factors for PGD  Intervention effectiveness  Intervention type: Non-specific | Family or friends   No specific cause/type of death |
| Laranjeira (2022) | To map and summarise findings from the existing literature regarding bereavement support interventions (i.e., psychosocial and psychotherapeutic interventions) for family carers of people who died of COVID-19 | Systematic review n=7 | Intervention effectiveness  Intervention type: Psychosocial & psychotherapeutic | Informal/family carers Adults  COVID-19 |
| Law (2019) | To review the needs of bereaved parents following the death of a young person from cancer and to inform the support they require from healthcare professionals from treatment centres | Systematic review n=16 | Grief and bereavement experiences | Parents  Health condition or terminal illness (cancer) |
| Lestienne (2021) | To examine the use and benefits of online resources dedicated to people bereaved by suicide | Systematic review n=12 | Grief and bereavement experiences  Intervention effectiveness/ implementation  Intervention type: Online or mobile | Non-specific relationship to the deceased  Suicide |
| Lichtenthal (2015) | To assess and appraise the literature on bereavement outcomes, follow-up, and needs to determine an evidence-based standard for routine assessment of bereavement needs of parents whose children died from cancer | Systematic review n=94 (including 1 integrated review) | Grief and bereavement experiences  Intervention effectiveness   Intervention type: Bereavement follow-up | Parents with family (siblings or grandparents)   Health condition or terminal illness (cancer) |
| Linde (2017) | To provide an overview of the current state of evidence concerning the effectiveness of interventions that focus on grief for people bereaved by suicide | Systematic review n=7 | Intervention effectiveness  Intervention type: Non-specific | Non-specific relationship to the deceased  Suicide |
| Logan (2018) | To explore bereaved, decedent, and respondent-related determinants of the provision of social support | Systematic review n=42 | General grief reactions  Grief and bereavement experiences | Non-specific relationship to the deceased  No specific cause/type of death |
| Lopez Perez (2022) | To determine the factors associated with complicated grief in the end-of-life phase within the palliative care context | Systematic review n=17 | Risk factors for PGD | Informal/family carers  Adults  Health condition or terminal illness (end of life) |
| Lord (2017) | To understand the experiences of staff supporting adults with intellectual disabilities with issues of death, dying and bereavement | Systematic review n=13 | Grief and bereavement experiences | Professionals - care workers for bereaved people who have a learning disability  No specific cause/type of death |
| Lundorff (2017) | To estimate the prevalence of prolonged grief disorder in the general adult bereaved population | Systematic review n=14 | Extent of PGD  Risk factors for PGD | Non-specific relationship to the deceased  Adults  non-violent death |
| Maass (2022) | To summarise the evidence of bereavement groups for symptoms of grief and depression | Systematic review n=14 RCTs | Intervention effectiveness  Intervention type: Bereavement groups | Non-specific relationship to the deceased  Adults  No specific cause/type of death |
| Mason (2020) | To describe risk and protective factors for complicated grief; examine current complicated grief interventions; and conduct a gap analysis of the interventions given the risk and protective factors | Systematic review n=32 | Risk factors for PGD  Intervention effectiveness  Intervention type: Non-specific | Informal/family carers  Adults; North America  Specific health condition or terminal illness |
| Mayland (2020) | To review and synthesise learning from previous literature focused on the impact on grief and bereavement during infectious disease outbreaks | Systematic review  n=6 (including 1 SR) | Grief and bereavement experiences | Non-specific relationship to the deceased  Adults  Health condition or terminal illness (pandemic or infectious disease) |
| Mayland (2021) | To review and synthesise the existing evidence on bereavement care, within the United Kingdom, for ethnic minority communities in terms of barriers and facilitators to access; models of care; outcomes from, and satisfaction with, service provision | Systematic review n=7 (including 1 narrative review) | Grief and bereavement experiences | Non-specific relationship to the deceased    UK ethnic minority populations  No specific cause/type of death |
| Mc Grath-Lone (2022) | To identify key messages from research concerning families’ experiences of perinatal loss and their perceptions of good practice that may be applicable to infant removal at birth | Review of reviews n=12 SRs | Grief and bereavement experiences  Intervention effectiveness  Intervention type: Non-specific | Defined parents with family (parents or family) UK or other high-income countries  Perinatal loss |
| McGill (2022) | To determine the feasibility, acceptability and effectiveness of brief contact interventions when delivered to people who have been bereaved and to describe the characteristics and outcomes of the interventions delivered and investigated to date | Systematic review n=15 | Intervention effectiveness  Intervention type: Brief contact | Non-specific relationship to the deceased  No specific cause/type of death |
| McNeil (2020) | To review the current literature on parental grief and bereavement in low- and middle-income countries | Systematic review n=11 (including 1 SR) | Grief and bereavement experiences  Intervention effectiveness  Intervention type: Non-specific | Parents Low- and middle-income countries  No specific cause/type of death |
| McNeil (2021) | To characterise the grief and bereavement experiences of fathers after the death of a child | Systematic review n=21 | Grief and bereavement experiences | Parents (fathers) Males  No specific cause/type of death |
| Meller (2019) | To examine the grief experiences of nurses who work in hospital settings after the death of a patient in their care and the factors that may impact nurses experiencing grief within the workplace | Systematic review n=5 | Grief and bereavement experiences | Professionals - nurses   No specific cause of death - specific setting (acute hospital settings) |
| Meunier (2021) | To review and synthesise the extent of scientific literature on the specific experiences of workers coping with perinatal loss and the resulting bereavement | Systematic review n=15 | Grief and bereavement experiences | Parents (working parents)  Perinatal loss |
| Morris (2019) | To review research on parental grief resulting from the death of a younger, typically dependent child in the paediatric range, and its lasting impact on parents | Systematic review n=42 | Extent of PGD  Risk factors for PGD  General grief reactions | Parents  No specific cause/type of death |
| Moss (2021) | To map bereavement interventions to established core outcomes for evaluating bereavement support among informal caregivers, and to identify grief support interventions that improve informal caregivers’ ability to cope with the grief | Systematic review n=3 RCTs | Intervention effectiveness  Intervention type: Non-specific (hospital settings) | Informal/family carers  No specific cause of death - specific setting (ICU) |
| Nielsen (2016) | To investigate key issues relating to anticipatory grief and preparedness for the death; definitions, measurement tools, and potential effects on outcomes for adult informal/family carers of adult terminally ill patients | Systematic review n=34 | Risk factors for PGD  General grief reactions | Informal/family carers Adults  Health condition or terminal illness (terminal illness, end of life) |
| O'Riordan (2022) | To undertake a systematic review of the existing literature on complicated grief in people with intellectual difficulties | Systematic review n=18 (including 4 literature reviews & commentaries) | Extent of PGD  Risk factors for PGD  Intervention effectiveness  Intervention type: Non-specific | Non-specific relationship to the deceased  Intellectual disability  No specific cause/type of death |
| Obst (2020) | To summarise and appraise the literature focusing on men’s grief following pregnancy loss and neonatal loss | Systematic review n=46 | Grief and bereavement experiences  General grief reactions | Parents (fathers) Males   Perinatal loss |
| Ogwulu (2015) | To explore the intangible costs of stillbirth in terms of their duration and economic implication | Systematic review n=20 | Grief and bereavement experiences | Parents High-income countries  Perinatal loss |
| Pachalla (2021) | To investigate the efficacy of bereavement support programs for siblings, extended family (other than parents), and community members after paediatric death | Systematic review n=4 | Intervention effectiveness  Intervention type: Non-specific | Siblings, extended family (not parents), or community members USA  No specific cause/type of death |
| Pan (2021) | To estimate the rate of complicated grief among Chinese people | Systematic review n=13 | Extent of PGD | Non-specific relationship to the deceased  China, Hong Kong or Taiwan  No specific cause/type of death |
| Paraiso Pueyo (2021) | To identify nursing interventions to help parents of neonates admitted to neonatal intensive care units cope with perinatal loss | Systematic review n=9 | Intervention effectiveness  Intervention type:  Non-specific (nursing) | Parents with family (immediate family)  Perinatal loss (neonatal ICU) |
| Parisi (2019) | To investigate the relationship between substance misuse and complicated grief | Systematic review n=12 | Extent of PGD  Risk factors for PGD  Intervention effectiveness  Intervention type: Non-specific | Non-specific relationship to the deceased  No specific cause/type of death |
| Parro-Jimenez (2021) | To review the status of the psychopathological research of complicated grief in adult population of Spain, specifically the prevalence and risk factors | Systematic review n=12 | Extent of PGD  Risk factors for PGD | Family or friends Adults; Spain  No specific cause/type of death |
| Patinadan (2022) | To review empirical interventions or interventional components that were observed to lessen or adaptively direct the experience of anticipatory grief for patients at the end of life and their family members | Systematic review n=10 RCTs | Intervention effectiveness   Intervention type: Non-specific - prevention (anticipatory grief) | Family or friends Adults  Health condition or terminal illness (end of life) |
| Pearce (2021) | To identify what works, how, and for whom, in the management of complicated grief in primary care | Systematic review n=42 (including 1 SR) | Grief and bereavement experiences | Family or friends Adults; high- or middle-income countries  No specific cause/type of death |
| Pelacho-Rios (2022) | To compile the most recently published interventions in bereavement support for parents who have lost a child, being particularly interested in those made from a meaning-centred approach | Systematic review n=21 | Intervention effectiveness  Intervention type: Non-specific | Parents with family  No specific cause/type of death |
| Pentaris (2022) | To review available empirical studies that explore the role of faith when grieving, among LGBTQIA+ individuals | Systematic review n=5 | Grief and bereavement experiences | Non-specific relationship to the deceased  LGBT+  No specific cause/type of death |
| Peters (2016) | To explore the meaningfulness of non-pharmacological care experienced by families throughout the experience of stillbirth from diagnosis onwards | Systematic review n=10 | Grief and bereavement experiences | Parents with family (siblings or grandparents)  Perinatal loss |
| Piil (2019) | To explore how informal caregivers of patients with primary malignant brain tumour (high-grade glioma) experience and manage their life situation after the death of the patient | Systematic review n=4 | Grief and bereavement experiences | Informal/family carers  Health condition or terminal illness (cancer - high-grade glioma) |
| Polita (2020) | To synthesise qualitative evidence about the bereavement experience of parents following the death of a child due to cancer | Systematic review n=14 | Grief and bereavement experiences | Parents  Health condition or terminal illness (cancer) |
| Purrington (2021) | To examine the available evidence on psychological adjustment to spousal bereavement in older adults | Systematic review n=15 | General grief reactions | Spouse Adults (65 years or older); western cultures in the Northern Hemisphere  No specific cause/type of death |
| Rait (2021) | To determine the effectiveness of bereavement interventions in reducing persisting psychological distress in bereaved family members after death in an adult ICU | Systematic review n=5 | Intervention effectiveness  Intervention type: Non-specific | Family or friends Adults  No specific cause of death - specific setting (ICU) |
| Raymond (2017) | To investigate nurses' roles and responsibilities in providing bereavement care during the care of dying patients within acute care hospitals | Systematic review n=7 | Grief and bereavement experiences | Professionals - nurses   No specific cause of death - specific setting (hospital - acute care) |
| Reime (2022) | To explore knowledge regarding professional helpers' experiences of providing assistance to people bereaved after a drug-related death | Systematic review n=no studies | Grief and bereavement experiences | Professionals - ‘professional helper'   Drug related deaths |
| Ridley (2020) | To describe the methods, structures, and procedures of bereavement care for children and adolescents after the loss of a sibling, and the impact on the families benefiting from these interventions | Systematic review n=23 | Intervention effectiveness/ implementation    Intervention type: Non-specific | Sibling Children or young people  Health condition or terminal illness (medical illness or unforeseen circumstances) |
| Riegel (2019) | To describe the practice of memory making as part of end-of-life care within an adult intensive care setting and determine reported outcomes from studies to date | Systematic review n=7 | Intervention effectiveness  Intervention type: Memory making | Family or friends   Health condition or terminal illness (end of life) |
| Roberts (2019) | To characterise the studies published in the last 5 years on grief interventions for bereaved older adults | Systematic review n=24 | Intervention effectiveness  Intervention type: Psychological (psychotherapy or psychotropic) | Non-specific relationship to the deceased  Adults (over 65 years old)  No specific cause/type of death |
| Roberts (2020) | To explore the lived experiences of surviving caregivers of parentally bereaved children | Systematic review n=14 | Grief and bereavement experiences | Parents or caregivers whose children were bereaved of a parent  No specific cause/type of death |
| Robinson (2019) | To examine the evidence for the usefulness of online peer support groups for bereaved persons | Systematic review n=9 | Intervention effectiveness  Intervention type: Online or mobile; peer support | Non-specific relationship to the deceased  No specific cause/type of death |
| Russ (2022) | To synthesise existing research on attachment patterns in adults experiencing complicated grief | Systematic review n=22 | Risk factors for PGD | Non-specific relationship to the deceased  Adults   No specific cause/type of death |
| Sajan (2022) | To identify recurrent themes and reactions of suicide survivors in their varying social interactions following the suicide and the impact of these social interactions on the grief process | Systematic review n=58 | Grief and bereavement experiences | Non-specific relationship to the deceased  Suicide |
| Sampson (2020) | To identify what we know about the relationship between continuing bonds and adaptation to bereavement | Systematic review n=16 | Risk factors for PGD  General grief reactions | Non-specific relationship to the deceased  No specific cause/type of death |
| Sanderson (2022) | To identify risk factors associated with complicated grief among family members of ICU decedents | Systematic review n=7 | Risk factors for PGD | Family or friends Adults   No specific cause of death - specific setting (ICU) |
| Schoonover (2022) | To clarify from the standpoint of bereaved parents what are considered helpful and unhelpful characteristics of informal support given by the support network of bereaved parents | Systematic review n=52 | Grief and bereavement experiences | Parents  No specific cause/type of death |
| Schoonover (2022) | To explore which support and accommodation strategies by the workplace are identified to be helpful versus unhelpful by bereaved parents in the workplace | Systematic review n=11 | Grief and bereavement experiences | Parents  No specific cause/type of death |
| Scott (2020) | To examine systematically whether there is an association between informal social support from family and friends after bereavement through sudden and/or violent causes and post-bereavement wellbeing | Systematic review n=16 | Risk factors for PGD  General grief reactions | Family or friends Adults  Violent or unnatural death |
| Seo (2020) | To review studies investigating the effect of bereavement care provided for parents in the neonatal intensive care unit | Systematic review n=5 | Intervention effectiveness  Intervention type: Non-specific (hospital settings) | Parents  Perinatal loss (Neonatal ICU) |
| Setubal (2021) | To identify and synthesise the literature on existent instruments specifically measuring the grieving process after any perinatal loss and to identify factors that could moderate grief reactions | Systematic review n=67 (39 for factors and 6 for interventions) | Risk factors for PGD  General grief reactions  Intervention effectiveness  Intervention type: Non-specific | Parents  Perinatal loss |
| Shakespeare (2019) | To perform a qualitative meta-summary of parents' and healthcare professionals' experiences of care after stillbirth in low- and middle-income countries | Systematic review n=34 | Grief and bereavement experiences | Parents and healthcare professionals  Low- and middle-income countries  Perinatal loss |
| Shaohua (2021) | To evaluate the effectiveness of psychosocial interventions in reducing depression, anxiety, and grief among parents after perinatal loss | Systematic review n=17 RCTs | Intervention effectiveness  Intervention type: Psychosocial | Parents Adults  Perinatal loss |
| Shariff (2017) | To identify challenges and facilitators that nurses experience in delivering bereavement support during and after sudden or unexpected death in Intensive Care Units | Systematic review n=15 | Grief and bereavement experiences | Professionals - nurses  Sudden or unexpected death (ICU or critical care) |
| Shields (2017) | To examine studies that investigate the grief process of those bereaved by suicide | Systematic review n=11 | Grief and bereavement experiences | Family or friends  Suicide |
| Shulla (2018) | To examine sex differences in internalised and externalised behaviour and PTSD symptoms related to grief during adolescence | Systematic review n=14 | Grief and bereavement experiences  General grief reactions | Child/sibling  Children and young people   No specific cause/type of death |
| Skantharajah (2022) | To explore the current state of knowledge toward grief and bereavement of informal caregivers of adult/geriatric patients in the hospice and palliative/end-of-life care realm within North America | Systematic review n=29 | Extent of PGD  Risk factors for PGD  General grief reactions  Grief and bereavement experiences | Informal/family carers  North America  Health condition or terminal illness (end of life) |
| Stiffler (2017) | To understand parental coping and healing after the loss of an infant | Systematic review n=3 | Grief and bereavement experiences | Parents with family (grandparents)  No specific cause/type of death |
| Taggart (2015) | To identify relevant and pertinent themes and interventions within the literature relating to childhood traumatic grief | Systematic review n=11 | Risk factors for PGD  Grief and bereavement experiences  Intervention effectiveness  Intervention type: Non-specific | Non-specific relationship to the deceased  Children and young people (aged 0 to 26 years); Sub-Saharan Africa  Violent or unnatural death |
| Talseth (2017) | To develop an interpreted synthesised understanding of responses of survivors of suicide loss to the suicidal death of a close person | Systematic review n=15 | Grief and bereavement experiences | Non-specific relationship to the deceased    Suicide |
| Thiemann (2021) | To identify, appraise and summarise the literature concerning the prevalence of prolonged grief disorder in older adults | Systematic review n=9 | Extent of PGD | Non-specific relationship to the deceased  Adults (65 years or older)  Any non-violent death |
| Thornton (2019)^a^ | To summarise and synthesise extant literature on memory making in bereavement care for parents who experience the death of a newborn | Systematic review n=25 | Grief and bereavement experiences | Parents  Perinatal loss |
| Thrower (2022) | To identify current interventions addressing the grief and bereavement experiences of informal caregivers of geriatric patients in the Canadian palliative/end-of-life care realm | Systematic review n=18 | Intervention effectiveness  Intervention type: Non-specific | Informal/family carers Canada  Health condition or terminal illness (terminal illness) |
| Titlestad (2021) | To identify and synthesise patterns in qualitative and quantitative studies that shed light on how family members experience drug death bereavement | Systematic review n=8 | Grief and bereavement experiences | Parents with family (child, sibling, spouse, partner or grandparent)  Drug-related death |
| Treml (2021) | To investigate definitions and measurement tools of pre-loss grief and preparedness for death, as well as the associations of both constructs with caregiver characteristics, pre-loss psychological aspects and post-loss adjustment among caregivers of people living with terminal cancer | Systematic review n=35 | Risk factors for PGD | Informal/family carers  Adults  Health condition or terminal illness (cancer) |
| Uphoff (2022) | To summarise available evidence on the effectiveness of interventions to promote and protect mental health relating to four key life events and transitions: pregnancy and early parenthood, bereavement, unemployment, and housing problems | Review of reviews:  n=18 SRs (12 synthesised) | Intervention effectiveness  Intervention type: Non-specific | Non-specific relationship to the deceased  Adults working-age (19 to 64 years)  No specific cause/type of death |
| van Denderen (2015) | To critically review the available evidence regarding psychopathology experienced by the homicidally bereaved | Systematic review n=8 (13 articles) | Extent of PGD | Family or friends Violent or unnatural death (homicide) |
| van Kempen (2022) | To provide insight into the content of follow-up conversations between bereaved parents and regular healthcare professionals (HCPs) in paediatrics and how parents and HCPs experience these conversations | Systematic review n=10 | Grief and bereavement experiences | Parents or healthcare professionals  No specific cause/type of death |
| Vedder (2022) | To establish the extent of scientific evidence on the role of loneliness in adjustment to bereavement in adulthood | Systematic review n=63 (20 reported grief) | Risk factors for PGD  General grief reactions  Intervention effectiveness  Intervention type: Psychological | Non-specific relationship to the deceased  Adults  No specific cause/type of death |
| Vig (2021) | To study prevailing accounts of bereaved parents following the death of a child aged 0 to 12 years | Systematic review  n=111 | Grief and bereavement experiences | Parents (and/or legal guardians)  No specific cause/type of death |
| Vrkljan (2019) | To examine the effectiveness of interventions that target the three most common transitions in later life, namely bereavement, retirement, and relocation | Systematic review n=7 RCTS | Intervention effectiveness  Intervention type: Non-specific | Spouse Adults aged 50 years or older  No specific cause/type of death |
| Wagner (2020) | To investigate the effectiveness of web-based bereavement interventions compared with control groups in reducing symptoms of grief in adults | Systematic review n=7 RCTs | Intervention effectiveness  Intervention type: Online or mobile  Psychological | Non-specific relationship to the deceased  Adults  No specific cause/type of death |
| Waller (2016) | To examine the relative proportion of descriptive, measurement and intervention research in grief counselling, and the quality and effectiveness of intervention studies | Systematic review n=126 (76 intervention) | Intervention effectiveness  Intervention type: Psychological (grief counselling) | Non-specific relationship to the deceased  Adults   No specific cause/type of death |
| Wang (2018) | To summarise the research literature on spousal bereavement and/or widowhood in late life, with an emphasis on immigrants to Western countries in general and older Chinese adults | Systematic review n=50 | General grief reactions  Grief and bereavement experiences | Spouse Adults (65 years or older); immigrants in Western countries and Chinese older adults in Mainland China or Hong Kong  No specific cause/type of death |
| Weiskittle (2018) | To critically evaluate the existent literature on the effectiveness of visual art modalities with the bereaved | Systematic review n=27 | Intervention effectiveness  Intervention type: Art/visual therapy | Non-specific relationship to the deceased  No specific cause/type of death |
| Williams (2020) | To understand the lived experiences of male partners during and after miscarriage, and to identify any support requirements | Systematic review n=22 (27 articles) | Grief and bereavement experiences | Parents (fathers) Males; high-income countries  Perinatal loss |
| Williams (2021) | To identify from the existing literature if physical activity can benefit grief outcomes in individuals who have been bereaved | Systematic review n=25 | Intervention effectiveness  Intervention type: Physical activity | Non-specific relationship to the deceased  No specific cause/type of death |
| Wilson (2017)^a^ | To synthesise the existing evidence regarding the impact of psychosocial interventions to assist adjustment to grief, pre- and post-bereavement, for family carers of people with dementia | Systematic review n=3 | Intervention effectiveness  Intervention type: Psychosocial | Informal/family carers  Health condition or terminal illness (dementia) |
| Wilson (2017) | To determine the state of bereavement services evaluation, to catalogue service types, and to identify which service or services, if any, demonstrate clear evidence of effectiveness | Systematic review n=38 | Intervention effectiveness  Intervention type: Structured/formal support | Non-specific relationship to the deceased  No specific cause/type of death |
| Wilson (2020) | To identify and consolidate contemporary evidence on: the incidence or prevalence of prolonged or persistent grief; and who develops it or is diagnosed as suffering from it, and correspondingly what causative factors or predictors are associated with prolonged or persistent grief | Systematic review n=11 | Extent of PGD  Risk factors for PGD | Non-specific relationship to the deceased  No specific cause/type of death |
| Wilson (2022) | To determine the extent of research on bereavement humour, and explore and describe the research evidence available at this time | Systematic review n=11 | Grief and bereavement experiences | Non-specific relationship to the deceased  No specific cause/type of death |
| Wojtkowiak (2020) | To analyse ritual in evidence-informed treatments for prolonged and traumatic grief; to learn what kind of ritual acts help in dealing with prolonged grief | Systematic review n=22 | Intervention effectiveness  Intervention type: Interventions that included ritual elements | Non-specific relationship to the deceased  No specific cause/type of death |
| Wray (2022) | To systematically identify and synthesise qualitative literature exploring support experiences of parentally bereaved children and surviving parents | Systematic review n=15 | Grief and bereavement experiences | Child and spouse (surviving parent)  No specific cause/type of death |
| Wright (2020) | To derive an understanding of how spirituality and perinatal bereavement intersect | Systematic review n=12 | Grief and bereavement experiences | Parents (mothers)  Females  Perinatal loss |
| Wright (2022) | To identify the unique needs of older adults after the loss of an adult child | Systematic review n=26 | Grief and bereavement experiences | Parents Adults (mean age 60 years or older)  No specific cause/type of death |
| Yan (2022) | To review the literature on grief and bereavement of family and friends following medical assistance in dying | Systematic review n=28 (Including 3 SRs and 3 reviews) | Risk factors for PGD  General grief reactions  Grief and bereavement experiences | Family or friends  Assisted Suicide/euthanasia |
| Yuan (2022) | To estimate the prevalence of prolonged grief disorder (PGD) and its symptoms amongst Chinese parents who lost their only child and cannot have a second child (Shidu parents) and identify subgroups at elevated risk for PGD | Systematic review n=7 | Extent of PGD  Risk factors for PGD | Parents  China  No specific cause/type of death |
| Ζavrou (2016) | To investigate the experience of people whose family member has died by suicide, with an emphasis on how they experience and make sense of the effects of the event, and the support they receive | Systematic review n=4 | Grief and bereavement experiences | Family or friends  Suicide |
| Zavrou (2022) | To synthesise qualitative data on the interpretation of loss in suicide-bereaved family members, their coping strategies and the impact on themselves and their family | Systematic review n=16 | Grief and bereavement experiences | Parent with family (spouses, children, or siblings)  Suicide |
| Zuelke (2021) | To conduct a systematic review and meta-analysis on the effectiveness and feasibility of internet- and mobile-based interventions against symptoms of grief after bereavement | Systematic review n=9 RCTs | Intervention effectiveness/ implementation   Intervention type: Online or mobile Psychological | Non-specific relationship to the deceased    Adults  No specific cause/type of death |

^a^These five reviews were included and coded based on the information in the title and abstract only as the full text was not readily available at the time of conducting the research.

NB: Details relating to the characteristics of the bereaved, restrictions on inclusion, and nature of death are based on reported eligibility criteria.

ICU=intensive care unit; LGBTQIA+=lesbian, gay, bisexual, transgender, queer, questioning, intersex, asexual or other; PGD=prolonged grief disorder; RCT=randomised controlled trial; SR=systematic review.
