# Supplementary material for: Grief, bereavement and prolonged grief disorder: scoping and mapping the evidence
Source: BJPsych Open. 2025 Jul 11;11(4):e149. doi: 10.1192/bjo.2025.10050 (PMC12247072; doi:10.1192/bjo.2025.10050)
Supplement: Raine et al. supplementary material 3 — Raine et al. supplementary material [file S2056472425100501sup003.docx]

## Supplementary file 3

Number of reviews by relationship to the deceased and cause/type or setting of death

| **Specific relationships (n=123)** | Specific health condition or terminal illness  (n=36) | Perinatal loss  (n=34) | Suicide  (n=20) | Violent or unnatural death (n=7) | Assisted suicide  (n=3) | Non-violent death  (n=2) | Drug related death (n=2) | Sudden or unexpected death (n=2) | COVID-19  (n=1) | Mass events  (n=1) | No cause of death -specific setting  (n=16) | No cause/type or setting of death  (n=88) |
| --- | --- | --- | --- | --- | --- | --- | --- | --- | --- | --- | --- | --- |
| Parents only (n=34) | **5** | **16** |  |  |  |  |  |  |  |  |  | **13** |
| Informal carers (n=18) | **14** |  |  |  | **1** |  |  |  | **1** |  | **2** |  |
| Parents with other family (n=15) | **1** | **5** | **2** |  |  |  | **1** |  |  |  | **3** | **3** |
| Child/sibling (n=13) | **4** |  | **2** |  |  |  |  |  |  |  |  | **7** |
| Professionals (n=11) |  |  | **1** |  |  |  | **1** | **1** |  |  | **4** | **4** |
| Spouse/partners (n=10) | **1** |  |  |  |  |  |  |  |  |  |  | **9** |
| Mothers (n=8) |  | **8** |  |  |  |  |  |  |  |  |  |  |
| Fathers (n=4) |  | **3** |  |  |  |  |  |  |  |  |  | **1** |
| Parents & health professionals (n=3) |  | **2** |  |  |  |  |  |  |  |  |  | **1** |
| Child & surviving parent (n=3) |  |  |  |  |  |  |  |  |  |  |  | **3** |
| Colleague (n=1) |  |  | **1** |  |  |  |  |  |  |  |  |  |
| Daughters (death of mother) (n=1) |  |  |  |  |  |  |  |  |  |  |  | **1** |
| Siblings, extended family or community (n=1) |  |  |  |  |  |  |  |  |  |  |  | **1** |
| Co-workers, family members, and close friends (n=1) |  |  |  |  |  |  |  | **1** |  |  |  |  |
| **All non-specific relationships (n=89)** | **11** |  | **14** | **7** | **2** | **2** |  |  |  | **1** | **7** | **45** |
